# The potent effect of mycolactone on lipid membranes

**DOI:** 10.1371/journal.ppat.1006814

**Published:** 2018-01-10

**Authors:** Milène Nitenberg, Anaïs Bénarouche, Ofelia Maniti, Estelle Marion, Laurent Marsollier, Julie Géan, Erick J. Dufourc, Jean-François Cavalier, Stéphane Canaan, Agnès P. Girard-Egrot

**Affiliations:** 1 Univ. Lyon, Université Lyon 1, CNRS, Institut de Chimie et Biochimie Moléculaires et Supramoléculaires, ICBMS—UMR 5246, GEMBAS team, Lyon, France; 2 Aix-Marseille Univ, CNRS, EIPL, Marseille, France; 3 CRCINA, INSERM, Université de Nantes, Université d'Angers, Angers, France; 4 Univ. Bordeaux, CNRS, Bordeaux INP, Chemistry and Biology of Membranes and Nano-objects, CBMN UMR 5248, Pessac, France; 5 Aix-Marseille Univ, CNRS, LISM, Marseille, France; McGill UniversityHealth Centre, CANADA

## Abstract

Mycolactone is a lipid-like endotoxin synthesized by an environmental human pathogen, *Mycobacterium ulcerans*, the causal agent of Buruli ulcer disease. Mycolactone has pleiotropic effects on fundamental cellular processes (cell adhesion, cell death and inflammation). Various cellular targets of mycolactone have been identified and a literature survey revealed that most of these targets are membrane receptors residing in ordered plasma membrane nanodomains, within which their functionalities can be modulated. We investigated the capacity of mycolactone to interact with membranes, to evaluate its effects on membrane lipid organization following its diffusion across the cell membrane. We used Langmuir monolayers as a cell membrane model. Experiments were carried out with a lipid composition chosen to be as similar as possible to that of the plasma membrane. Mycolactone, which has surfactant properties, with an apparent saturation concentration of 1 μM, interacted with the membrane at very low concentrations (60 nM). The interaction of mycolactone with the membrane was mediated by the presence of cholesterol and, like detergents, mycolactone reshaped the membrane. In its monomeric form, this toxin modifies lipid segregation in the monolayer, strongly affecting the formation of ordered microdomains. These findings suggest that mycolactone disturbs lipid organization in the biological membranes it crosses, with potential effects on cell functions and signaling pathways. Microdomain remodeling may therefore underlie molecular events, accounting for the ability of mycolactone to attack multiple targets and providing new insight into a single unifying mechanism underlying the pleiotropic effects of this molecule. This membrane remodeling may act in synergy with the other known effects of mycolactone on its intracellular targets, potentiating these effects.

## Author summary

Buruli ulcer is a necrotizing skin disease caused by an environmental mycobacterial pathogen. The pathogenesis of this neglected tropical disease involves the production of a toxin, mycolactone, which spreads through the tissues, away from the infecting organisms. Mycolactone has pleiotropic effects on fundamental cellular processes, resulting in pronounced cytotoxicity and immunosuppressive effects that together drive progressive ulceration. The molecular mechanisms underlying its cellular effects have been partly deciphered, but multiple cellular targets have been identified. A literature survey revealed that most of the identified targets of mycolactone are membrane receptors residing in particular domains of the plasma membrane. Despite its lipid-like nature, mycolactone has been shown to be intracellular, implying that it can cross the plasma membrane. We describe here a surprising membrane-reshaping effect of mycolactone due to effects on lipid domain formation. By reversing lateral lipid segregation, mycolactone may disrupt the formation of domains with well-established roles in the regulation of cellular signaling pathways. This remodeling of the cell plasma membrane may underlie the molecular events enabling mycolactone to attack multiple targets.

## Introduction

Buruli ulcer (BU) is the third most common human mycobacterial infection in the world, after tuberculosis and leprosy [1,2]. BU is a neglected tropical disease of the skin and subcutaneous tissue caused by an environmental pathogen, *Mycobacterium ulcerans* (*M*. *ulcerans*). This disease, which can affect all age groups and both sexes, is commonest in West Africa and parts of Australia, but has been reported in over 30 countries worldwide [3,4]. These painless ulcers affect at least 5,000 patients per year and are thought to be heavily underreported [5]. Infection with *M*. *ulcerans* results in persistent severe necrosis with no acute inflammatory response. The ulcer begins as a painless nodule or papule on the skin, which, if left untreated, progresses to massive ulceration that may cover 15% of the skin of the patient, resulting in significant morbidity [5,6]. BU is not lethal, but patients may suffer lifelong disfigurement, functional impairment and disability unless the infection is recognized and treated at an early stage.

*M*. *ulcerans* pathogenesis is mediated by a necrotizing immunosuppressive toxin, mycolactone (S1 Fig). This lipid-like polyketide macrolide has been identified as the main virulence factor produced by *M*. *ulcerans* and is responsible for the skin lesions and tissue necrosis [6,7]. After its production [8,9], this diffusible toxin is excreted in vesicles derived from the bacterial membrane and enriched in extracellular matrix, which acts as a reservoir of the toxin [10]. *In vitro*, mycolactone has been shown to localize in the cytosol of cultured murine fibroblasts, through non-saturable and non-competitive uptake in the presence of excess mycolactone [11,12]. Mycolactone has also been reported to accumulate in a time- and dose-dependent manner in the cytoplasm of human epithelial cells and lymphocytes, but not in the plasma or nuclear membranes of the cell [13]. These findings suggest that mycolactone can diffuse across cell membranes by non-cell-specific passive diffusion to reach its intracellular targets [11,14].

Mycolactone A/B (a 3:2 ratio of *Z-/E*-isomers of the C4’-C-5’ bond in the long “Southern” polyketide side chains, S1 Fig), which is produced by the most virulent strains of *M*. *ulcerans*, has been shown to have pleiotropic effects on fundamental cellular processes, such as cell division, cell death and inflammation, depending on toxin dose and exposure time [14,15]. Exposure to pure mycolactone is cytotoxic for many cell lines, but the dose and exposure required for cell death are highly variable [15]. Early studies on cell lines suggested a role for mycolactone in cell-cycle arrest in the G_1_/G_0_ phase and apoptosis [7,16]. However, recent studies have suggested that anoïkis, due to cytoskeleton rearrangements, leading to changes in cell adhesion and detachment, is a much more likely mechanism of cell death *in vivo* [3,17]. By inducing changes to the cytoskeleton and disrupting tissue structure, this toxin compromises cell structure and homeostasis through the impairment of extracellular matrix biosynthesis [18]. In addition to its cytotoxicity, mycolactone has immunosuppressive activity, resulting in a lack of local inflammation despite extensive tissue damage, together with inhibition of the local immune response [19–23]. At low concentrations, this molecule has been found to be a powerful analgesic, due to its stimulatory effect on the angiotensin receptor [24]. These effects may account for the painlessness of BU lesions. The precise molecular mode of action of mycolactone in eukaryotic cells remains unclear, but a number of cellular targets have been identified. A literature survey revealed most of these targets to be membrane receptors residing in ordered plasma membrane nanodomains known to modulate the functionalities of membrane proteins [25,26].

Mycolactone can impair the migration of naïve T cells to peripheral lymph nodes [27], where they make contact with antigen-presenting cells during T-cell receptor activation. This alteration of T-cell homing is accompanied by a decrease in L-selectin receptor (CD62-L) levels. The downregulation of this receptor normally involves proteolytic cleavage upon stimulation, but the cleavage of L-selectin seems to involve membrane microdomains, which act as a signaling platform [28]. Similarly, the chemokine receptors involved in T-cell inflammatory responses also reside in membrane domains and, the depletion of cholesterol from membranes decreases chemokine binding and abolishes chemokine receptor signaling [29].

Another effect of mycolactone A/B is hyperactivation of the Src-family kinase, leading to the depletion of intracellular calcium and a downregulation of T-cell receptor (TCR) expression, limiting the T-cell response to stimulation and potentially contributing to apoptosis [3,14]. This hyperactivation is initiated by the relocalization of Lck in the microdomains of the plasma membrane, triggered by the action of the toxin [30]. Mycolactone has been reported to inhibit angiotensin II binding, in a dose-dependent manner, and to elicit signaling through human type 2 angiotensin II receptors (AT_2_Rs), leading to a potassium-dependent hyperpolarization of neurons, accounting for the painlessness of BU lesions [24]. AT_2_R, like AT_1_R, is a G protein-coupled receptor (GPCR). Microdomains (both lipid rafts and caveolae) have been reported to be involved in regulating GPCR signaling, by affecting both signaling selectivity and coupling efficacy [31,32].

Mycolactone has recently been shown to modulate Wiskott-Aldrich syndrome protein (WASP) and neural WASP (N-WASP), two members of a family of scaffold proteins that transduce various endogenous signals in dynamic remodeling of the actin cytoskeleton [17]. In immune cells, WASP regulates ordered lipid domain dynamics during immunological synapse formation, which involves clustering of the microdomains of the plasma membrane for optimal T-cell activation. WASP, which is recruited to lipid domains immediately after TCR stimulation, is required for the movements of these microdomains [33]. By disrupting WASP autoinhibition [17], mycolactone can hijack actin-nucleating factors, leading to uncontrolled activation of the ARP2/3-mediated assembly of actin, and a deregulation of lipid domain dynamics. Similarly, mycolactone provokes a disruption of the protein C anticoagulant pathway, with a depletion of thrombomodulin (TM) receptors at the surface of endothelial cells [34]. Nevertheless, the receptors of the protein C activation and activated protein C (APC) signaling pathways are colocalized in the lipid microdomains of endothelial cells [35,36].

Finally, it has recently been reported that mycolactone inhibits the function of the Sec61 translocon [37–39], a transmembrane channel located in the endoplasmic reticulum (ER) membrane [40]. This ubiquitous complex is responsible for cotranslational protein translocation, a universally conserved process in the biosynthesis of secretory and membrane proteins that operates for most of the 30–50% of mammalian proteins carrying a canonical signal peptide [41]. In investigations of transmembrane proteins (TNF), monotypic proteins (COX-2) and conventionally secreted proteins (IL-6), Hall *et al*. showed that mycolactone prevents ER protein translocation, with the proteins concerned being translated in the cytosol, where they are marked for rapid destruction by the proteasome. In this way, mycolactone causes a selective ~30% decrease in membrane-associated proteins and prevents the production of the vast majority of N-glycosylated proteins [37,38]. Cholesterol and sphingolipid levels are lower in the ER than in the plasma membrane and other organelles, but it has been suggested that ER membranes nevertheless contain lipid domains [42,43]. The fractionation of rough ER integral membrane proteins with 0.18% Triton X-100 (similar to the treatment of cytoplasmic membranes with 1% Triton X-100, which has successfully revealed the presence of lipid domains in the cytoplasmic membrane) showed that the 0.18% Triton X-100 fraction contained mostly ER-resident proteins, including, in particular, the Sec61alpha subunit [44], the central transmembrane component of the sec 61 ER translocon targeted by mycolactone [38,39,45].

Thus, mycolactone has diverse complex effects on a range of cells and tissues, and the underlying mechanism unifying its pleiotropic effects seems to be its action through microdomain-associated proteins [14]. In this study, we aimed to characterize in more detail the effects of pure mycolactone on biological membranes, focusing, in particular, on the effects of this toxin on microdomain segregation. Indeed, no molecular-scale description of the effects of this toxin on the cell plasma membrane before it reaches its cellular targets, most of which are located in the ordered plasma membrane nanodomains, has ever been reported.

We investigated the capacity of mycolactone to interact with membranes and its effects on lipid organization when crossing the membrane, with several biophysical techniques, including Langmuir monolayers, which we used as an *in vitro* model of cell membranes, together with fluorescence and Brewster angle microscopy. Langmuir monolayers consist of supramolecular lipid films that form at an air-buffer interface. They can mimic biological membranes and are, thus, attractive membrane models, because the thermodynamic relationship between monolayer and bilayer membranes is direct, and monolayers overcome, independently of their lipid composition, the limitations associated with the regulation of lateral lipid packing encountered in model bilayer systems [46]. They are widely used in studies of peptide or membrane probe/lipid interactions [47–52], and in studies of membrane-protein association [53–61]. Brewster angle microscopy (BAM), which was used for the *in situ* characterization of Langmuir monolayers, provides additional information about membrane morphology and lipid organization at the air-water interface [62–64]. In our system, we used a lipid composition closely resembling that of the plasma membrane, including among others, 33% sphingomyelin (SM) and 19% cholesterol (Chol), which was considered to be a biologically normal concentration (natural membranes contain 5–50 mol% cholesterol [65,66]). Using this experimental approach, we demonstrated marked effects of mycolactone on membranes, and were able to visualize, for the first time, the capacity of this molecule to disrupt membranes at the molecular level.

## Results

We investigated the interaction of mycolactone with biological membranes and evaluated the possible influence of lipid composition (*i*.*e*., with or without cholesterol), by exploring the binding properties of the toxin with a model membrane reconstituting lipid monolayers at the air/water interface. These so-called Langmuir monolayers are half-membrane models [46], and they can be used not only to characterize protein–membrane interactions, but also to determine the mechanism of action of bioactive molecules on cell membranes. We chose this model for study on the basis of its simple experimental design, the possibility of changing lipid composition easily and its suitability for evaluations of the membrane insertion capacity of membranotropic molecules [47–61,67].

### Interfacial behavior of mycolactone at an air/buffer interface

We characterized the surfactant properties of mycolactone, by evaluating its interfacial behavior at the air/buffer interface and in the absence of lipids.

Experiments without lipids at the air/buffer interface can be used to determine: i) the concentration at which amphiphilic molecules saturate the lipid-free interface (*i*.*e*., surface saturation concentration) and ii) the concentration range minimizing aggregation and, therefore, useful for experiments. It is widely accepted that the analytical concentrations to be injected into the subphase for subsequent molecule/lipid interaction analyses should be based on such pre-evaluations and lower than the surface saturation concentration, to prevent artifacts due to molecule aggregation [51,67–69].

**Fig 1:**
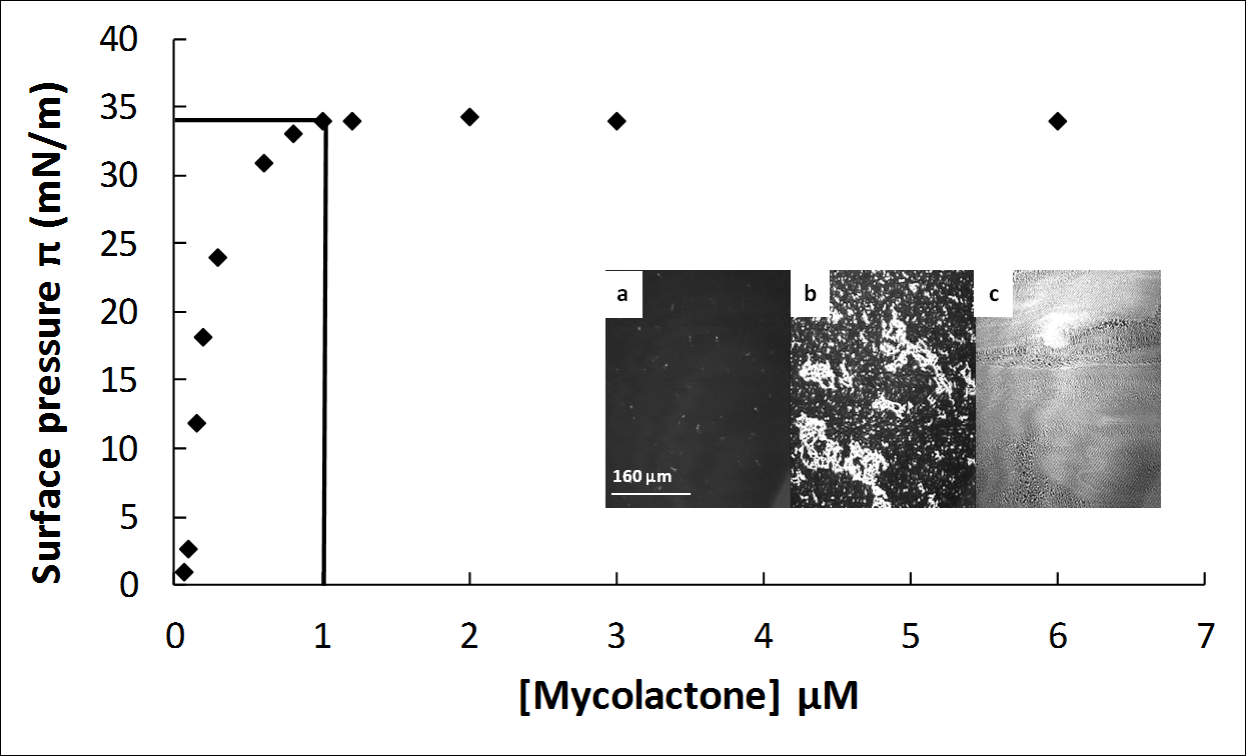
Adsorption of mycolactone at the air/buffer interface. Equilibrium surface pressures (π_e_) reached at the end of the adsorption kinetics for different concentrations of mycolactone injected into the subphase (PBS pH 7.4). Each point corresponds to the mean value of three kinetic experiments. The surface saturation concentration (1 μM) was determined at the start of the plateau. **Inset**: BAM images at equilibrium surface pressure (π_e_), after the injection of mycolactone at a final concentration of 0.6 μM (a), 1.2 μM (b) or 6 μM (c). Image scale: 483 × 383 μm^2^. All measurements were repeated at least three times for each concentration, with a satisfactory reproducibility, and the mean values are presented here.

The surface saturation concentration was determined by tensiometry [70,71]. Various mycolactone concentrations, from 60 nM to 6 μM, were injected into the PBS subphase. For each concentration, the adsorption of mycolactone at the air/buffer interface was monitored by continuous surface pressure measurement until the equilibrium value, π_e_, was reached. The curve of π_e_ as a function of mycolactone concentration rapidly increased to reach a plateau at 34 mN/m (Fig 1). At this surface pressure, the interface was saturated with mycolactone molecules, regardless of the concentration of the toxin in the subphase. The surface saturation concentration of mycolactone was then determined at the start of the plateau, and was found to be 1 μM.

Brewster angle microscopy (BAM) images recorded at π_e_ with a final mycolactone concentration of 0.6 μM (a), 1.2 μM (b) or 6 μM (c) (Fig 1, Inset) confirmed the ability of this molecule to accumulate in a concentration-dependent manner at the air/PBS (pH 7.4) interface, and to form a very thick film (3.7 ± 0.3 nm thick) at very high concentrations (6 μM). At a concentration of 0.6 μM, below the apparent surface saturation concentration of 1 μM, mycolactone formed a homogeneous interfacial film, but with some bright nuclei also visible (spots, Fig 1, Inset a). These bright nuclei resulted from aggregate formation, as demonstrated by dynamic light scattering (DLS) for mycolactone solutions in PBS pH 7.4 in S1 Appendix.

Thus, mycolactone displayed surfactant properties at a nude interface. To prevent the association of molecules into aggregates in the next experiments, we used a low concentration of mycolactone, 60 nM. At this concentration, mycolactone interacts with lipids as a monomer (see S1 Appendix).

### Influence of cholesterol on the packing and stability of the membrane-mimicking monolayers

The aim of this study was to analyze the interaction of mycolactone with biological membranes. We studied two membrane models: i) a monolayer consisting of a lipid mixture resembling that of the plasma membrane (given in mol%) [72–75]: 39% POPC, 33% SM, 9% POPE, 19% Chol (mixture 1), and ii) a monolayer with the same lipids but without cholesterol (given in mol%): 48% POPC, 41% SM, 11% POPE (mixture 2). Cholesterol is known to regulate lipid segregation in plasma membranes [25,26]. Mycolactone receptors have been reported to be located in ordered plasma membrane microdomains. We therefore investigated the effects of this particular membrane lipid on the ability of mycolactone to bind to membranes.

We first studied the interfacial properties of the two monolayers alone, and the impact of cholesterol on lipid organization in particular, at 20 and 25°C.

### Effect of cholesterol on surface pressure (π)—molecular area (A) isotherms

**Fig 2:**
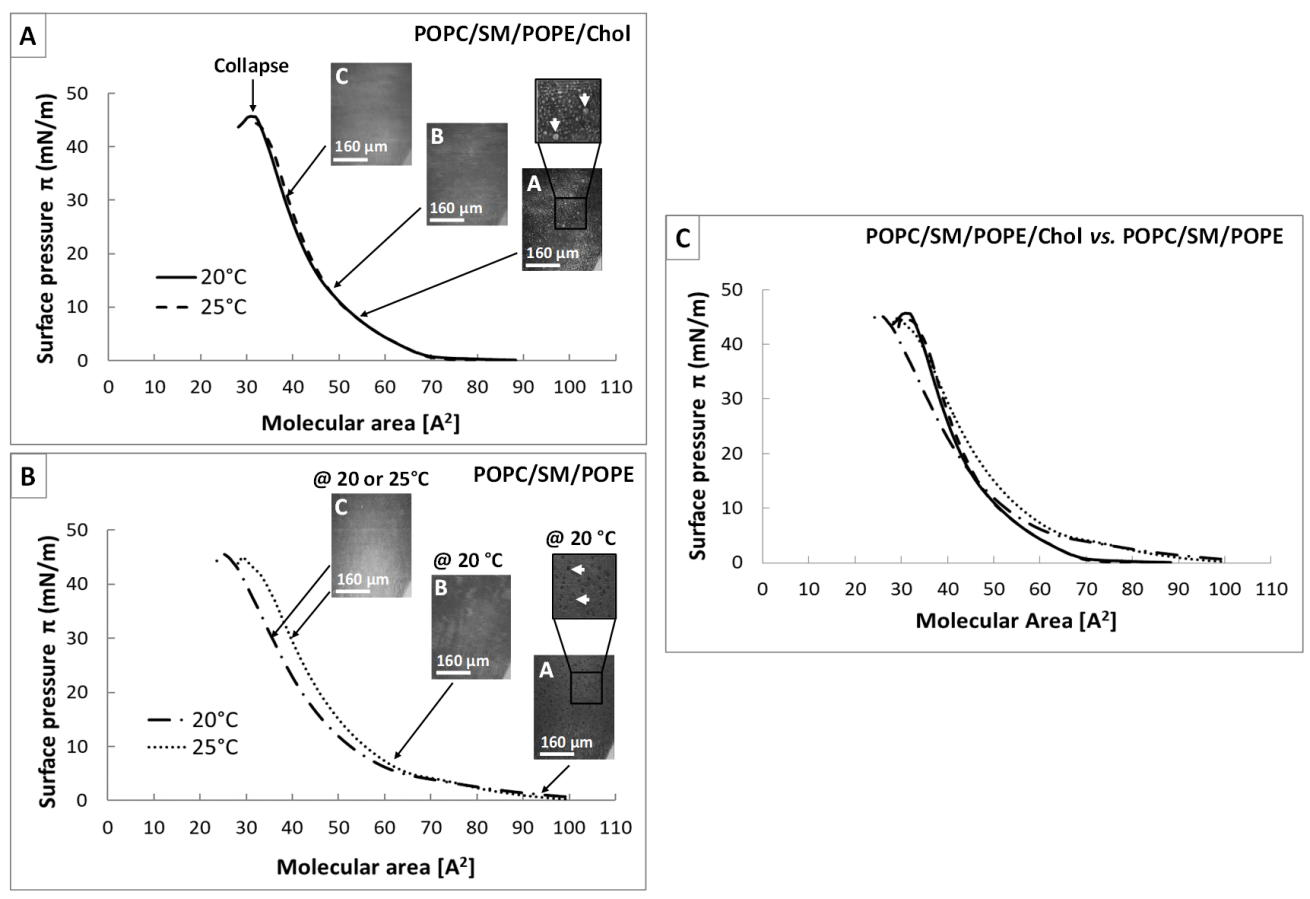
Surface pressure (π)–molecular area (*A*) isotherms and corresponding BAM images of monolayers with and without cholesterol. (A) π-*A* isotherms of mixture 1 (39% POPC, 33% SM, 9% POPE, 19% Chol given in mol%) recorded at 20°C (solid line) or 25°C (dashed line). (B) π-*A* isotherms of mixture 2 (48% POPC, 41% SM, 11% POPE given in mol%) recorded at 20°C (dashed-dotted line) or 25°C (dotted line). (C) Comparison of isotherms of the above-mentioned monolayers. Isotherms were recorded on PBS subphase (pH 7.4). Each isotherm corresponds to the mean of three experiments. BAM images were recorded during compression of the monolayer at a constant rate of 0.045 nm^2^.molecule^-1^.min^-1^. Images A and B were recorded at 20°C. The images obtained for C were identical for 20 and 25°C. The estimated error for monolayers is ±0.05 mN/m for π and ≤ 0.01 nm^2^ for (A). Image scale: 483 × 383 μm^2^.

Whatever the temperature, the π-*A* isotherms of mixture 1 (Fig 2A) showed the monolayer to be in liquid-condensed (LC) phase throughout compression. The beginning of the steep rise started at a molecular area of 70 Å^2^, and the monolayer was compressed up to a lateral pressure of π_coll_ = 45 mN/m, corresponding to collapse. The molecular area at collapse, A_coll_, was 31 Å^2^. This area was smaller than expected for two fatty acyl chains of phospholipids; the area per CH_2_ chain in a close-packed configuration is approximately 20 Å^2^ [76]. This discrepancy can be explained by the condensing effect of the cholesterol. The molecular area of a pure expanded monolayer of POPC (the major component of mixtures 1 and 2) at a lateral pressure of π_coll_ = 40 mN/m is ~40 Å^2^ at 20 or 25°C, consistent with the T_m_ value (-4°C) of POPC (S2 Fig). The addition of 19% cholesterol to the POPC monolayer, resulted in the same A_coll_ for the 81% POPC/19% cholesterol mixture at 25°C, but this area decreased to ~32 Å^2^ at 20°C. This suggests that the presence of 19% cholesterol lead to extensive condensation of the POPC monolayer in the liquid-expanded state (S2 Fig). Cholesterol has been shown to dehydrate lipid bilayers, resulting in lipid condensation [77]. The much lower level of condensation observed in the presence of mixture 1 (~37 Å^2^ at π = 40 mN/m) in terms of the area of POPC (~40 Å^2^) may be due to the presence of 9% POPE in mixture 1, at least partly preventing the condensing effect of cholesterol. BAM images recorded during compression revealed that the small lipid domains (shown in light gray) present at the start of compression (Fig 2A, image A, white arrows) increased in size and coalesced (Fig 2A, image B) to form a homogeneous interfacial film at the end of compression (Fig 2A, image C), regardless of temperature. This observation is consistent with the behavior of a condensed monolayer. Finally, the monolayer was homogeneous at 30 mN/m (Fig 2A, Image C), the lateral surface pressure reported for biological membranes [78].

A different pattern was observed for the isotherms of mixture 2 (Fig 2B), with the monolayers displaying a phase transition at both temperatures. Surface pressure began to increase at a higher molecular area, 98 Å^2^. BAM images taken at 20°C revealed the presence of holes (grayscale level identical to the background) in a continuous lighter phase (Fig 2B, image A, white arrows) for surface pressures below 3 mN/m. These holes gradually disappeared during compression until a short plateau was reached at about 5 mN/m. Beyond this point, the monolayer was homogeneous (Fig 2B, image B) and in a liquid-condensed state until collapse (π_coll_ = 45 mN/m; *A*_coll_ = 25 Å^2^). At 25°C, the phase transition, which could be attributed to the liquid-expanded/liquid-condensed (LE/LC) transition phase of monolayers incorporating SM [79,80], was attenuated. Consequently, the monolayer remained homogeneous (absence of holes at low surface pressures) throughout compression (Fig 2B, image C), until collapse (π_coll_ = 44 mN/m; *A*_coll_ = 30 Å^2^). In the absence of cholesterol, no lipid domains were observed in the monolayers. The apparent condensing effect observed for mixture 2 (S2 Fig) relative to the pure monolayer of POPC in the expanded state is due to the presence of 41% SM, a high-melting lipid (Tm = 41.4°C). The shift in *A*_coll_ values observed when the temperature was increased from 20 to 25°C could be explained, in all cases, by the disordering effect of the higher temperature on acyl chain packing, tending to fluidize the monolayer.

Superimposition of the isotherms recorded at 20 and 25°C (Fig 2C) highlighted the effect of cholesterol on the condensation state of the monolayer. At surface pressures below 10 mN/m, the isotherms of mixture 1 (with cholesterol) were shifted towards lower molecular areas than those of mixture 2 (without cholesterol). By contrast, at high surface pressures (above 25–30 mN/m), the isotherm of mixture 1 at 20°C was shifted towards larger areas than those of mixture 2 at the same temperature. This clear difference between the two mixtures was consistent with the modulation of membrane fluidity by cholesterol, through modification of the ordering of lipid acyl chains [81]: cholesterol tends to condense fluid phases (*i*.*e*., it increases the lipid chain ordering of the liquid-crystalline disordered phase) and to fluidize condensed phases (*i*.*e*., it decreases the lipid chain ordering of the solid-ordered phase) [82–85]. A comparison of the four isotherms also revealed that, in the absence of cholesterol at 20°C, the monolayer was extremely condensed. This condensation state may be directly due to the presence of 41% SM in mixture 2. Indeed, sphingolipids generally form a solid gel phase and are fluidized by sterols, which interact preferentially with them in the membrane [66,86]. Furthermore, the domains observed in mixture 1 (Fig 2A, image A) were probably characteristic of the liquid-ordered phase resulting from a ternary mixture of a high chain-melting lipid (like SM) and a low chain-melting lipid (like POPC) with cholesterol, and preferential interactions between Chol and SM [65,79,82,87–92].

### Stability of model lipid monolayers at a working surface pressure of 30 mN/m

We analyzed the interaction of mycolactone with monolayers at a working surface pressure of 30 mN/m, to mimic the lateral pressure of biological membranes [46,78]. As a control, and to decipher the effect of mycolactone more effectively, we checked the stability over time of the mixed monolayers in the presence of ethanol, the solvent used for mycolactone. For this purpose, we injected a volume of ethanol equivalent to that used for mycolactone solution (4.45 μL) into the subphase underneath the stabilized monolayer at 30 mN/m. We then recorded changes in surface pressure over a period of about seven hours. At 20°C, the monolayers were highly stable, with only small surface pressure variations (± 2 mN/m) over time (S3 Fig). At 25°C, a greater variation of surface pressure was observed (from ‒2 to ‒5 mN/m), possibly due to subphase evaporation.

**Fig 3:**
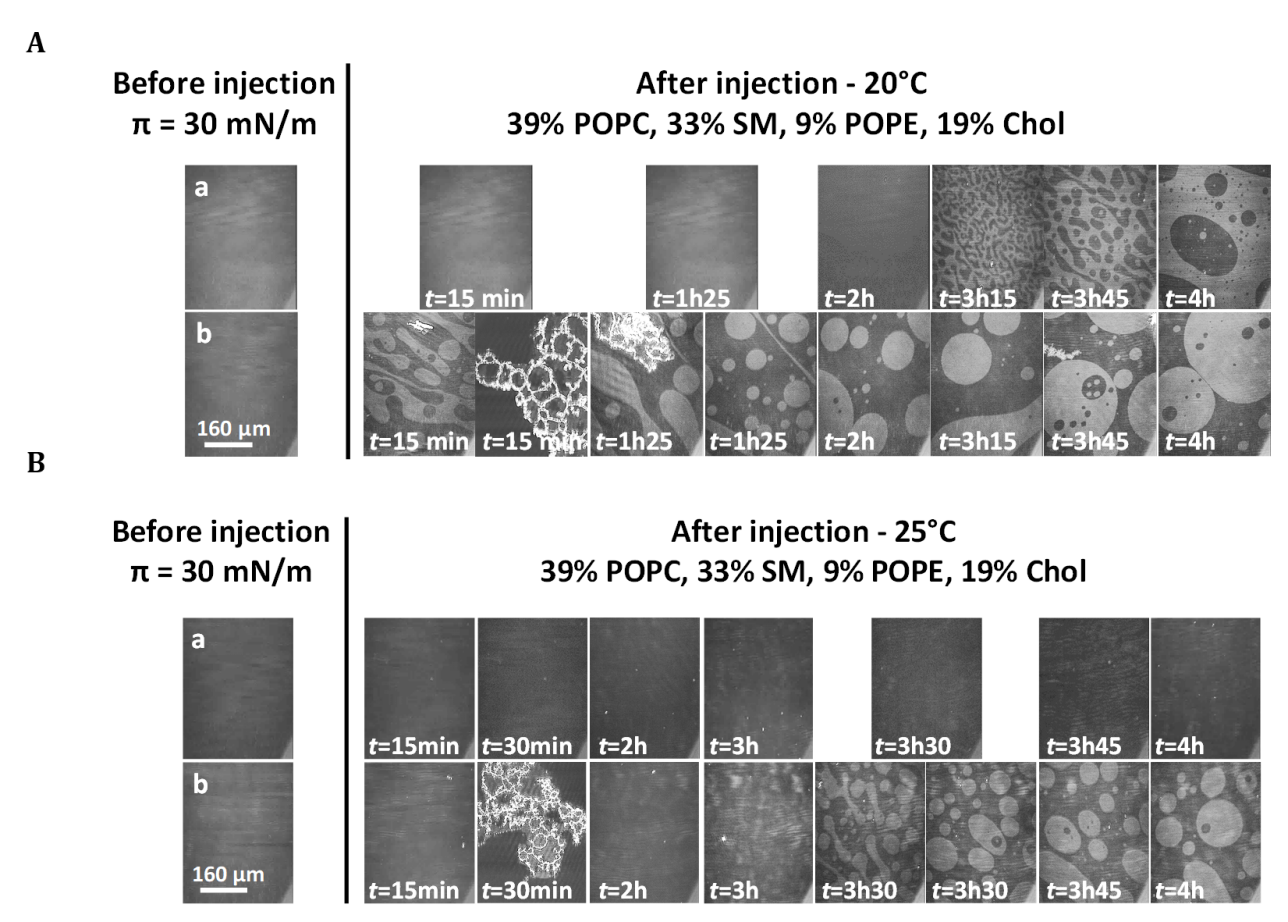
Effect of mycolactone on the lipid organization of mixed monolayers in the presence of cholesterol. BAM images of 39% POPC, 33% SM, 9% POPE, 19% Chol (mixture 1) monolayers after the injection (4.45 μL) of ethanol (row a) or mycolactone (row b) into the PBS subphase (pH 7.4) beneath the interfacial film compressed at an initial surface pressure of 30 mN/m at 20° (A) or 25°C (B). The injection was performed after a relaxation time of one hour, with the surface area kept constant (mobile barriers were stopped). The final concentration of mycolactone was 60 nM. Image scale: 483 × 383 μm^2^.

**Fig 4:**
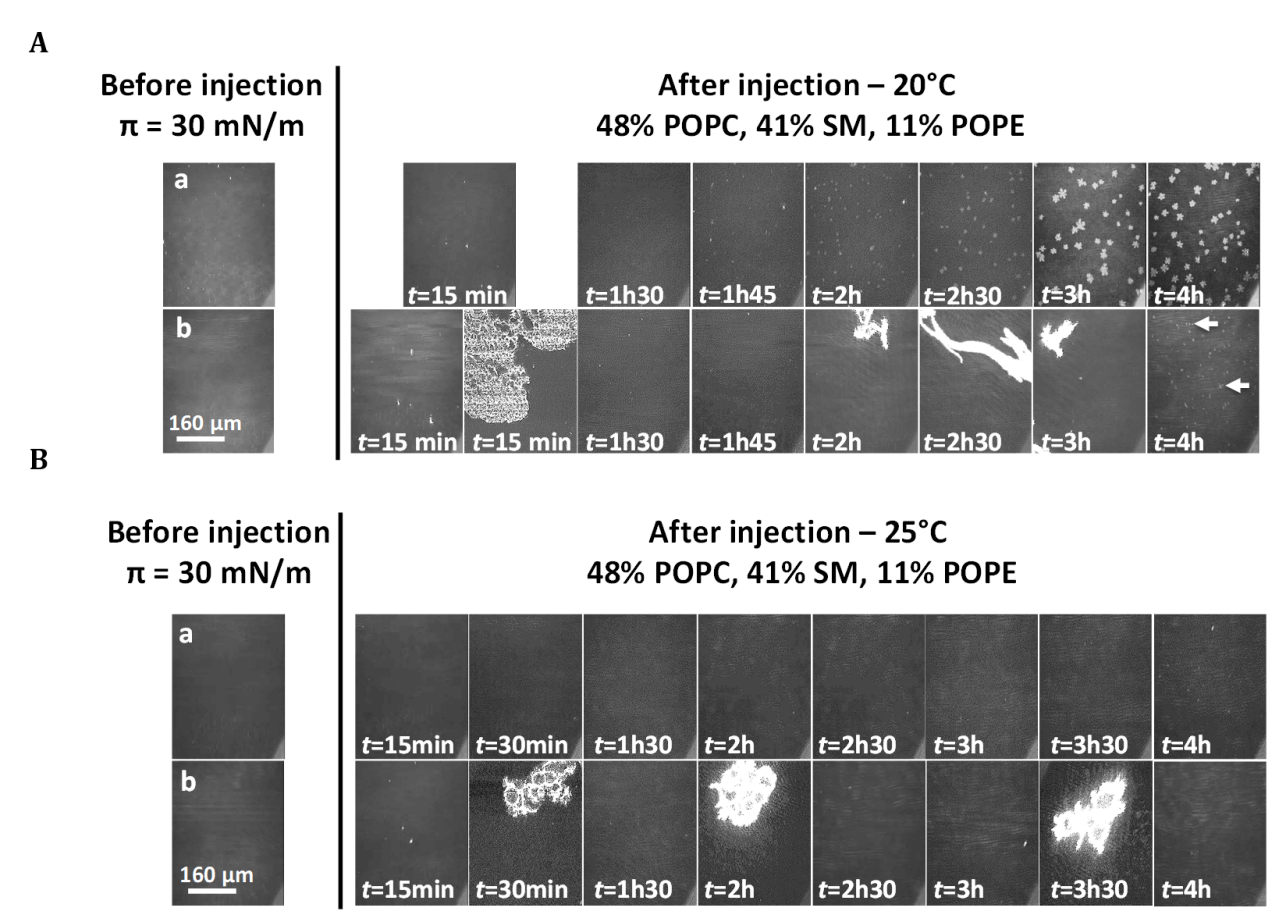
Effect of mycolactone on the lipid organization of mixed monolayers without cholesterol. BAM images of 48% POPC, 41% SM, 11% POPE (mixture 2) monolayers after the injection (4.45 μL) of ethanol (row a) or mycolactone (row b) in the PBS subphase (pH 7.4) beneath the interfacial film compressed at an initial surface pressure of 30 mN/m at 20°C (A) or 25°C (B). The injection was performed after a relaxation time of one hour, with the surface area kept constant (mobile barriers were stopped). The final concentration of mycolactone was 60 nM. Image scale: 483 × 383 μm^2^.

We used BAM images for simultaneous characterization of the morphology and lipid organization of the mixed monolayers at 20°C (Figs 3A and 4A, rows a) and 25°C (Figs 3B and 4B, rows a). All the monolayers were homogeneous after one hour of relaxation, just before injection. After injection, the changes in monolayer morphology differed between temperatures and membrane lipid compositions.

At 20°C, the mixed films displayed a phase segregation that differed according to the presence or absence of cholesterol. In the presence of cholesterol (Fig 3A, row a), lipid organization gradually changed, after about 3 h, with the formation of circular domains of an expanded fluid phase (dark phase) trapped within a more condensed phase (white phase). The same change in monolayer morphology was obtained without the injection of ethanol (S4 Fig, row a). This segregation, observed at 20°C, and leading to a new thermodynamic equilibrium with no loss of stability, could therefore be attributed to preferential interactions between cholesterol and the high-melting lipid SM in the mixed monolayer [65,82,83,87], with an expulsion of low-melting lipids such as POPC/POPE, resulting in the formation of round domains of fluid phase, as already reported for ternary mixtures of PC/SM/Chol [81,88,89]. In the absence of cholesterol, ordered domains appeared earlier, from the start of the experiment, and progressively grew in the form of “stars” (bright clusters, Fig 4A, row a). These domains resembled the condensed domains observed in the liquid-expanded/liquid-condensed (LE/LC) transition phase during the compression of a pure monolayer of SM on a PBS subphase (pH 7.4) at 20°C (S5 Fig). These findings suggest that SM molecules retain their ability to segregate over time in the mixed monolayer, but only in the absence of cholesterol.

Conversely, no segregation occurred at the higher temperature. Indeed, at 25°C, in the presence (mixture 1 –Fig 3B, row a) or absence (mixture 2 –Fig 4B, row a) of cholesterol, the two monolayers remained homogeneous throughout the entire experiment.

### The interaction of mycolactone with mixed monolayers modifies lipid segregation in membranes

We investigated the membrane-binding properties of mycolactone and evaluated the effect of this interaction on lipid organization in mixed films, by injecting a solution of mycolactone in ethanol into the subphase at a final concentration of 60 nM, beneath the monolayers of mixture 1 (with cholesterol) or mixture 2 (without cholesterol), compressed at an initial surface pressure π_i_ of 30 mN/m.

**Fig 5:**
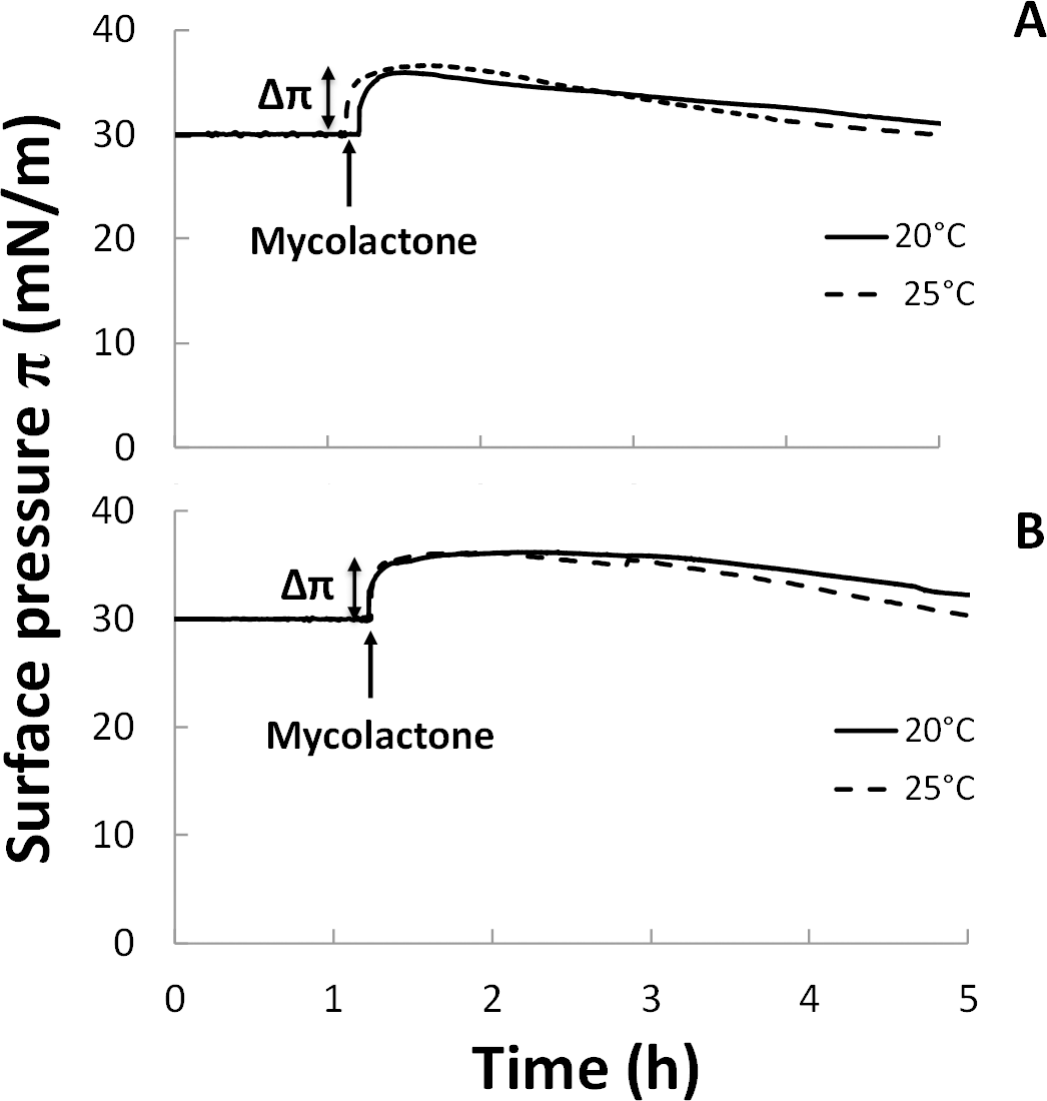
Interaction of mycolactone with mixed monolayers in the presence or absence of cholesterol. Adsorption kinetics (π-*t*) curves of mycolactone on monolayers composed of (A) mixture 1 (39% POPC, 33% SM, 9% POPE, 19% Chol) or (B) mixture 2 (48% POPC, 41% SM, 11% POPE) at 20°C (solid line) or 25°C (dashed line). Mycolactone was injected (4.45 μL) into the PBS subphase (pH 7.4) beneath the monolayer compressed at an initial surface pressure π_i_ of 30 mN/m after a relaxation time of one hour (arrows). Surface area was kept constant during the run. The final concentration of mycolactone was 60 nM. Each measurement was performed at least three times for each condition, and a representative curve is presented here.

Upon injection, regardless of lipid composition and temperature, the interaction of mycolactone with the monolayer resulted in a rapid increase in surface pressure up to ~36 mN/m within the first 15–20 minutes (Fig 5). After a stabilization period of about 1–1.5 h, π gradually decreased over time. The absence of cholesterol clearly did not affect the ability of mycolactone to penetrate into the monolayer; it simply delayed the decrease in surface pressure.

BAM images were taken before and after mycolactone injection, throughout the adsorption period (Figs 3 and 4, rows b). In all cases, monolayers were homogeneous at the initial surface pressure of 30 mN/m. Mycolactone injection modified lipid segregation in the monolayers independently of temperature, but differently according to the presence or absence of cholesterol in the monolayers.

For mixture 1 at 20°C (Fig 3A, row b), lipid organization changed rapidly over the first 15 min towards the formation of circular domains in a more condensed state (light gray phase), trapped within a less condensed phase (dark gray phase). This reorganization is essentially the opposite of the organization observed with the pure monolayer (Fig 3A, row a). A similar pattern was observed if the mycolactone was injected at the apparent saturation concentration of 1 μM (S4 Fig, row b). The time required for mycolactone to reverse the segregation pattern in membranes (15 minutes) corresponds to the time required for toxin penetration into the monolayer until stabilization. At 25°C (Fig 3B, row b), this segregation pattern occurred 3 h after injection, whereas no segregation was observed for the control monolayer at 25°C. Highly luminous structures (Fig 3, row b, *t* = 15 min or 85 minutes at 20°C and *t* = 30 min at 25°C), similar to those observed for mycolactone at the air/buffer interface (Fig 1, image b) were also observed. This feature indicated the presence of the toxin within the monolayer in the presence of cholesterol.

For mixture 2 at 20°C, no star-shaped domains were visible, contrasting with observations for the monolayer alone. Very small domains (small bright dots) became visible much later, 4 h after mycolactone injection (Fig 4A, row b, white arrows). At 25°C, no significant change in the morphology of the monolayer relative to the control was observed upon mycolactone injection (Fig 4B). Again, only the presence of bright objects corresponding to mycolactone at different time points (Fig 4B, rows b) attested to the interaction of the toxin with the monolayer. In the absence of cholesterol, the presence of the toxin within the monolayer therefore prevented SM molecules from aggregating, thereby fluidizing the condensed and extremely rigid mixture 2 monomolecular film (Fig 2B). At 25°C, the monolayer was fluid enough to prevent SM aggregation, and this attenuated the potential fluidizing effect of the mycolactone.

### Influence of cholesterol on the membrane-binding properties of mycolactone

We analyzed the influence of lipid organization on the membrane-binding properties of the toxin further, by investigating the effect of initial surface pressure on the interaction of mycolactone with the monolayers. For this purpose, we monitored the maximal increase in surface pressure Δπ_max_ immediately following toxin injection at various π_i_ values, ranging from 5 to 30 mN/m. This relationship has been widely used to assess lipid-protein interactions and to distinguish between electrostatic and hydrophobic interactions [53,55,57,58,60,61,68,93].

**Fig 6:**
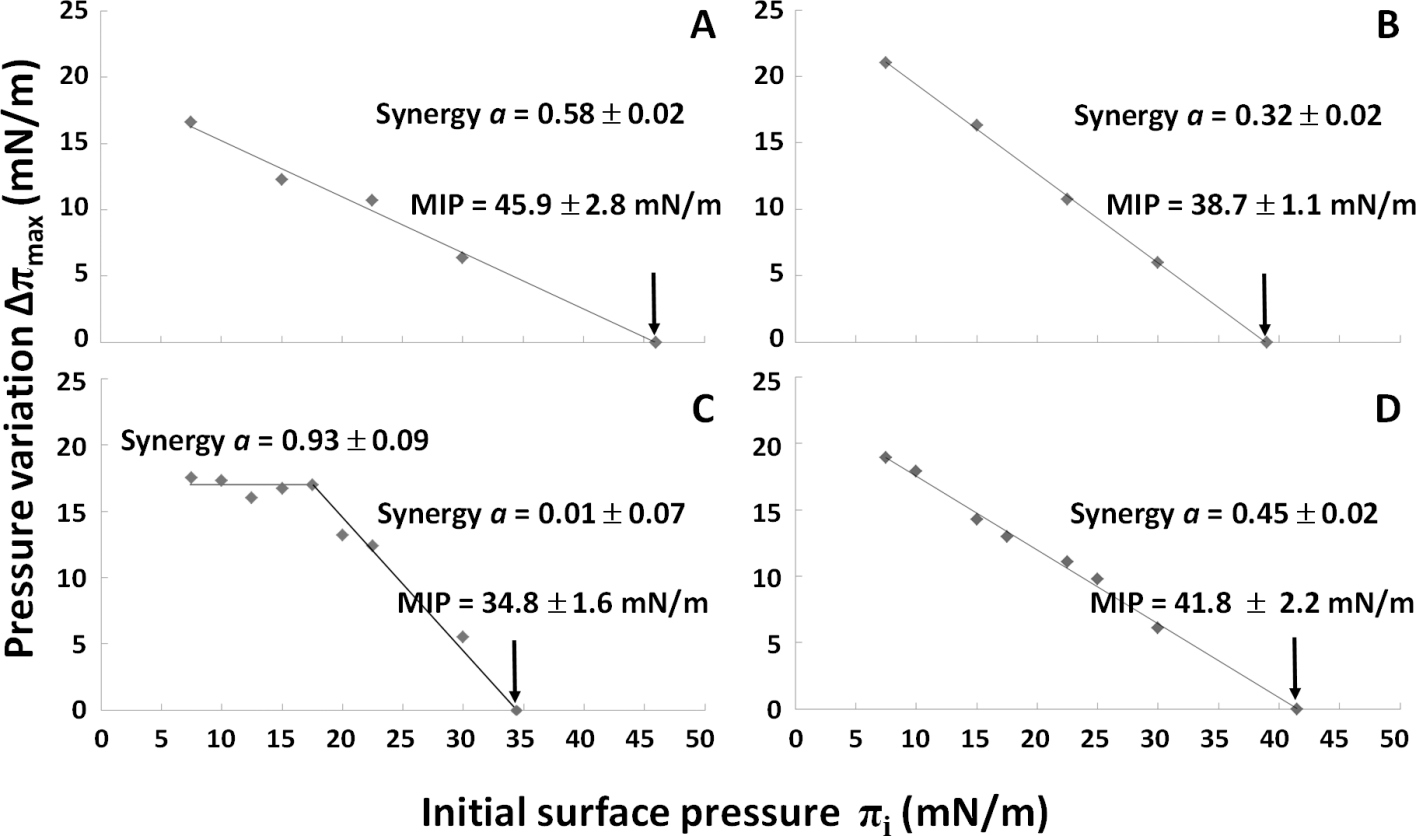
Influence of lipid packing on the membrane-binding properties of mycolactone. Change in surface pressure (Δπ, mN/m) when mycolactone interacts with mixed monolayers at different initial surface pressures (π_i_, mN/m). The nature of the lipid membrane was as follows: (A) and (B) mixture 1 consisting of 39% POPC, 33% SM, 9% POPE, 19% Chol. (C) and (D) mixture 2 consisting of 48% POPC, 41% SM, 11% POPE (given in mol%). Experiments were performed at 20°C (A) and (C), or 25°C (B) and (D). Each point corresponds to an independent measurement with a new lipid monolayer formed on PBS subphase (pH 7.4). The final concentration of mycolactone was 60 nM. Representative data from two or three independent assays are shown.

The Δπ_max_ = *f*(π_i_) plot shown in Fig 6 was used to evaluate the binding parameters of mycolactone on both types of Langmuir monolayers. Linear extrapolation to an increase in surface pressure of zero (Δπ_max_ = 0) can be used to determine i) the maximum insertion pressure (MIP), reflecting the influence of initial lipid packing density on the ability of the molecule to penetrate into the monolayer, and ii) the synergy factor "*a*" [50,56,93,94]. This factor, first described by Salesse *et al*. [56,94], provides insight into the mechanisms governing the interaction with lipid monolayers. A positive *a* value indicates favorable interactions, as further demonstrated by MIP values exceeding the estimated membrane lateral pressure (~30 mN/m). A null synergy factor reveals a stationary state, with no favoring or disfavoring of membrane binding. A negative synergy factor indicates unfavorable binding to the monolayer, corresponding to a repulsion of the molecule as a function of the compactness of the monolayer. Here, MIP and *a* provided useful information about the effect of lipid composition on the ability of mycolactone to interact with membranes.

For mixture 1, pressure variation profiles were similar at the two temperatures, with a linear decrease as a function of initial surface pressure π_i_. MIP and the *a* synergy factor were above 30–35 mN/m and positive, respectively, at both temperatures. These findings are consistent with strong insertion/penetration into the interfacial film and favorable interactions between mycolactone and the monolayer (Fig 6A and 6B). Furthermore, both MIP and *a* values were higher at 20°C (MIP = 45.9 ± 2.8 mN/m; *a* = 0.58 ± 0.02) than at 25°C (MIP = 38.7 ± 1.1 mN/m; *a* = 0.32 ± 0.02), suggesting that, in the presence of cholesterol, decreases in temperature leading to a rigidification of the monolayer may favor the interaction of mycolactone with the mixed film.

By contrast, the curve profiles for mixture 2 differed considerably between temperatures. At 20°C (Fig 6C), the plot obtained was split into two distinct phases: an initial plateau, for which Δπ_max_ remained constant at π_i_ values below 17.5 mN/m, with a synergy factor of 0.93 ± 0.09, and a second phase in which Δπ_max_ decreased at π_i_ values greater than 17.5 mN/m, associated with an MIP value of 34.8 ± 1.6 mN/m and a synergy factor close to 0 (*a* = 0.01 ± 0.07). Such ‘biphasic’ behavior was recently reported by Hädicke and Blume for the binding of small cationic peptides to anionic phospholipid monolayers [95], and may be related to the physical state of the monolayer. As shown by these authors, incorporation, through hydrophobic interactions, into the loosely packed monolayer in the LE phase can lead to a constant Δπ value, depending on the nature of the lipids making up the monolayer. By contrast, in the LC phase, Δπ and π_i_ display an inverse linear relationship, due to lipid condensation. For mixture 2, the monolayer displayed a LE/LC phase transition, as shown on the isotherms (Fig 2B), with the presence of holes in the loosely packed monolayer, as revealed by BAM (Fig 2B, image A). We therefore suggest that behavior similar to that proposed for small hydrophobic peptides may account for the unusual results obtained with mycolactone. Indeed, this toxin is also a small hydrophobic molecule (MW: 743.021), and, when it penetrates into a loosely packed monolayer in the LE phase by hydrophobic interactions, it triggers no increase in surface pressure because the monolayer is too weakly compressed (loosely packed) and the molecular area of the toxin is too small to cause lipid condensation at the interface. In addition, mycolactone was probably able to fill the space, *i*.*e*., the holes observed in the monolayer, due to its own surface activity, leading to an absence of surface pressure variation (Δπ_max_ remained constant) as long as the monolayer was weakly compressed.

Beyond 17.5 mN/m, the monolayer was sufficiently tightly packed to attain its condensed state (observed on the isotherm Fig 2B), yielding a negative slope of the Δπ_max_ = *f*(π_i_) plot (Fig 6C, part 2), with a synergy factor close to 0 (*a* = 0.01 ± 0.07). This value indicates that, even in a stationary state in which mycolactone was able to penetrate the monolayer at 20°C, no specific interactions (either favorable or unfavorable) occurred between mycolactone and lipids. The decrease in the ability of the molecule to penetrate the membrane was therefore entirely due to the physical condensation of the monolayer as a result of the increase in lipid packing density during compression [56,94].

At 25°C (Fig 6D), the curve profile and the MIP (41.8 ± 2.2 mN/m) were similar to those obtained for mixture 1 at the same temperature, but the *a* value (0.45 ± 0.02) was different. At the higher temperature (25°C *vs*. 20°C), the monolayer was more fluid, as revealed by the shift of the π-*A* isotherm towards larger molecular areas due to the disordering effect of the higher temperature on acyl chain packing (Fig 2B), and favorable interactions occurred between the toxin and the monolayer. As previously observed for π-*A* isotherms (Fig 2A and 2B), the effect of temperature on monolayer fluidity was more pronounced for mixture 2. This difference may account for the difference in synergy values.

At 25°C, the interaction of mycolactone with monolayers seems to be governed by greater monolayer fluidity. However, the presence of cholesterol in the monolayer enabled the mycolactone to penetrate into a more condensed monolayer. Indeed, if mycolactone insertion were regulated solely by monolayer fluidity (as observed at 25°C), then its insertion should increase with temperature, which was found to be the case in the absence (Fig 6C and 6D), but not in the presence of cholesterol (Fig 6A and 6B). Conversely, the binding parameters (MIP and synergy) were highest at 20°C in the presence of cholesterol (Fig 6A). As the synergy factor measures sensitivity to lipid acyl chain packing, we can conclude that the insertion of mycolactone into the monolayer was favored by the presence of cholesterol at lower temperatures, which favored increases in monolayer rigidity.

### Mycolactone reverses the lipid organization of the liquid-ordered (Lo) phase in membranes

We investigated the effects of mycolactone on lipid segregation in the monolayer in the presence of cholesterol, by performing the same experiments (π_i_ = 30 mN/m, 20°C) by fluorescence microscopy (FM), with mixture 1 labeled with TopFluor Cholesterol probe. This molecular probe can be used to study intracellular cholesterol dynamics, because its diffusion in the plasma membrane is free and unhindered [96]. In this context, the fluorescent domains observed were, thus, those containing the TopFluor Cholesterol molecule. In our study, the use of this fluorescent marker made it possible to track the localization of cholesterol in the monolayer and its distribution in domains.

**Fig 7:**
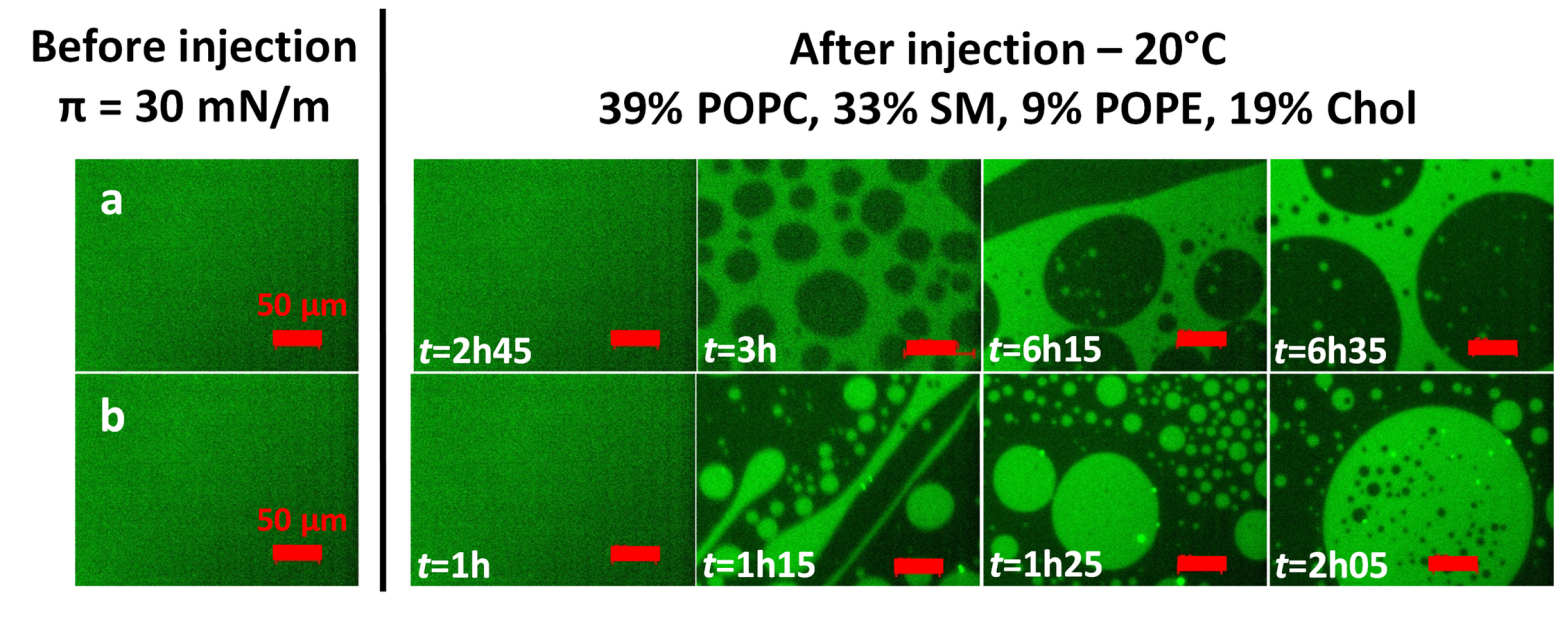
Influence of mycolactone interaction on the distribution of the liquid-ordered (L_o_) phase in the membrane. Fluorescence images of monolayers consisting of 39% POPC, 33% SM, 9% POPE, 19% Chol and including 0.5% BODIPY-cholesterol (TopFluor Cholesterol), after the injection (4.45 μL) of ethanol (row a) or mycolactone (row b) into the PBS subphase (pH 7.4) beneath the interfacial film compressed at an initial surface pressure of 30 mN/m at 20°C. The injection was performed after a relaxation time of one hour, and surface area was kept constant (mobile barriers were stopped). The final concentration of mycolactone was 60 nM. Scale bar: 50 μm.

Lipid segregation in the control monolayer was observed 3 h after ethanol injection (4.45 μL), with the appearance of circular dark domains trapped within a light phase (Fig 7, row a). Segregation occurred more rapidly in the presence of the toxin (in 1h15), but with a pattern opposite to that in the control, with the formation of circular fluorescent domains within a dark phase (Fig 7, row b). In ternary mixtures, SM is known to interact preferentially with cholesterol to form domains of liquid-ordered (L_o_) phase, corresponding to a phase intermediate between the liquid-condensed phase (L_c_) and the fluid liquid-expanded (L_e_) phase [91,92]. It can therefore be inferred from our measurements that the green fluorescent areas correspond to domains of liquid-ordered phase, whereas the dark areas correspond to domains of fluid phase [96]. Thus, these FM experiments yielded results identical to those obtained with BAM for the pure monolayer or after the injection of mycolactone (Fig 3A), in support of our conclusion. A similar correlation between the results of BAM and FM was reported in another recent study [79].

By modifying the interactions between SM molecules, and, probably, between SM and cholesterol, through physical insertion in the monolayer (the toxin impeded the segregation of SM in mixture 2 at 20°C without specific interaction, *a* = 0), mycolactone reversed the segregation of the Lo phase in the monolayer. Thus, mycolactone probably interacts preferentially with the Lo phase, which is intermediate between the highly condensed phase of SM and the fluid phase of POPC. This conclusion is consistent with the results obtained for the two mixtures at 20°C, and with the synergic interaction observed only in the presence of cholesterol (*a*>0 for mixture 1 at 20°C–Fig 6A). This preferential interaction with the Lo phase may also explain why the presence of cholesterol enhanced the penetration capacity of mycolactone at lower temperatures, which were associated with lower levels of fluidity (highest MIP and *a* value for mixture 1 at 20°C–Fig 6A).

### Mycolactone has a detergent-like membrane-reshaping activity

The adsorption kinetic (π-*t*) curves showed a progressive decrease in surface pressure after the injection of mycolactone, at both temperatures (Fig 5). This suggests that the toxin affects monolayer stability and may have a detergent-like effect. Indeed, detergents are amphiphilic molecules with surfactant properties that can solubilize lipids, depending on membrane phase and composition [97].

**Fig 8:**
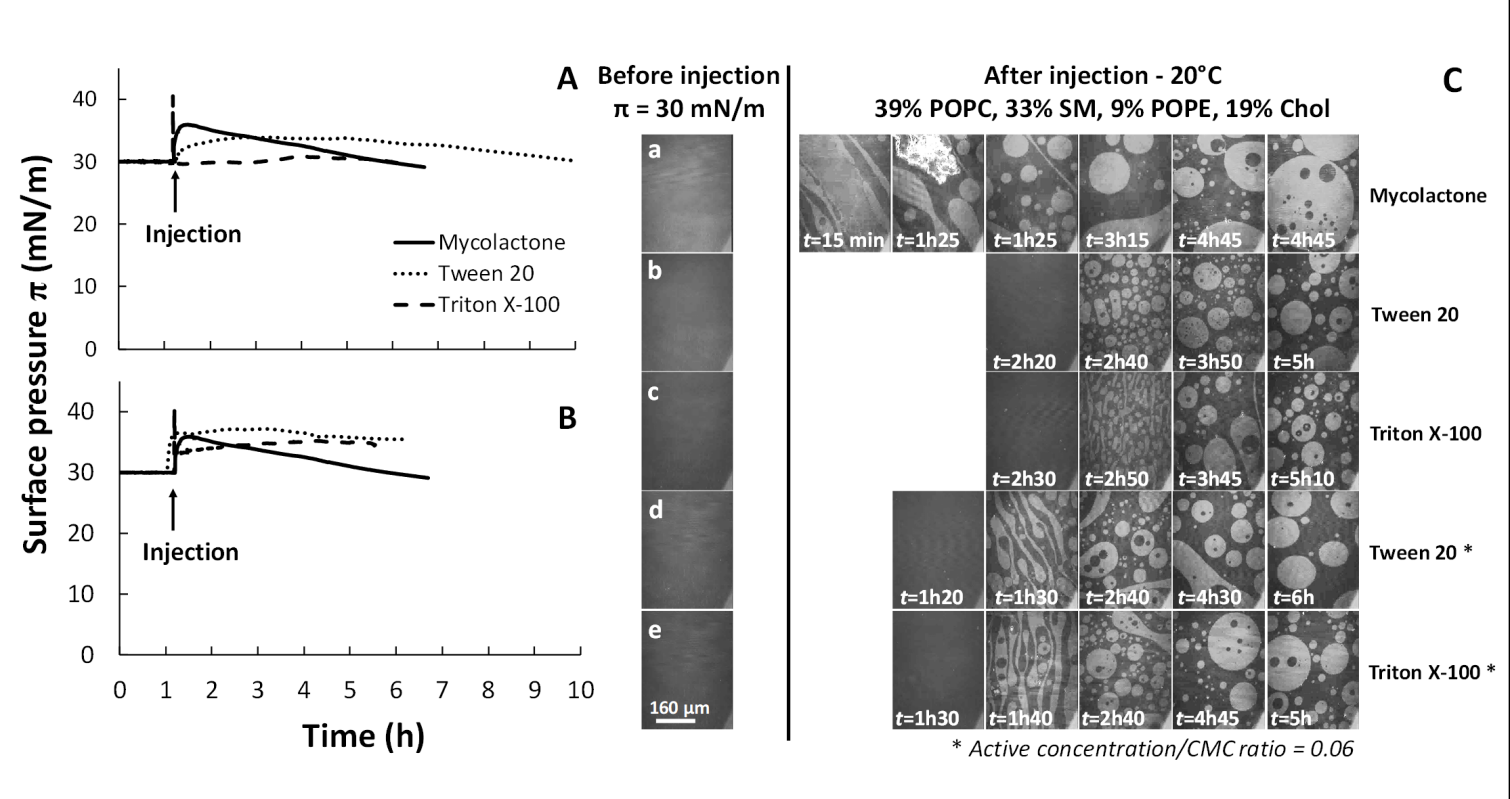
Detergent effect on mixed monolayers in the presence of cholesterol. Adsorption kinetics (π-t) curves of detergent on monolayers consisting of 39% POPC, 33% SM, 9% POPE, 19% Chol (mixture 1) at 20°C, and the corresponding BAM images. (A) Tween 20 or Triton X-100 was injected into the PBS subphase (pH 7.4) beneath the monolayer at a final concentration of 60 nM. (B) Tween 20 or Triton X-100 was injected into the PBS subphase (pH 7.4) beneath the monolayer at a constant final “Active concentration/CMC ratio” of 0.06. (C) BAM images corresponding to the adsorption kinetics (π-t) curves after the injection of Tween 20 (b) and (d), or Triton X-100 (c) and (e), at a constant final concentration of 60 nM (b) and (c), or an “Active concentration/CMC ratio” of 0.06 (d) and (e). (a) Images recorded after the injection of a 60nM mycolactone (final concentration). In all experiments, the monolayer was compressed at an initial surface pressure π_i_ of 30 mN/m and detergents were injected after a relaxation time of one hour (arrows). Surface area was kept constant during the run. Each measurement was performed at least three times for each condition, and a representative curve is presented here. Image scale: 483 × 383 μm^2^.

We tested this hypothesis, by performing interaction assays with a final concentration of 60 nM Tween 20 or Triton X-100 (*i*.*e*., non-ionic detergents) at 20°C, with a monolayer of mixture 1 compressed at a π_i_ of 30 mN/m. Tween 20 is known to solubilize lipid membranes regardless of their aggregation state [98], whereas Triton X-100 solubilizes the liquid-disordered (L_d_) phase but not the liquid-ordered phase (L_o_) [81,88,97,99]. The kinetic curves recorded were then compared with that obtained with mycolactone (Fig 8A).

When Tween 20 was injected beneath the monolayer, the surface pressure π increased to a plateau value of about 34 mN/m, gradually decreasing thereafter (Fig 8A). Investigations of monolayer morphology by BAM (Fig 8C, row b) revealed that, upon interaction, lipid organization shifted towards the formation of circular condensed domains (white phase) trapped within a fluid phase (dark phase), as in the case of mycolactone (Fig 8C, row a). However, this lipid reorganization occurred 2h40 after detergent injection, later than for the toxin (Fig 8C, row b).

The surface pressure π remained constant after Triton X-100 injection (Fig 8A). The absence of an effect on the surface pressure stability of mixture 1 was therefore compatible with an absence of L_o_ phase solubilization (a property of Triton X-100). However, BAM images revealed that Triton X-100 provoked the same pattern of lipid segregation as mycolactone (condensed domains in a fluid phase), but with the same time-shift (2h50) as for Tween 20 (Fig 8C, row c). Similar changes in the morphology of monolayers containing SM and cholesterol upon interaction with Triton X-100 have already been reported [100], and this detergent, which can induce L_o_/L_d_ phase segregation in a typical raft-like ternary mixture, was recently described as a potent membrane-reshaping agent [97,99].

All these findings reveal, therefore, that the presence of 60 nM detergent or toxin in the subphase modifies lipid segregation in the POPC/SM/POPE/Chol monolayer in a manner opposite to that in the control.

In these experiments, both the detergents and the toxin were injected into the subphase at the same final concentration (60 nM). However, the solubilizing action of a detergent depends on its critical micellar concentration (CMC) and on the detergent/membrane ratio at which it is used [101]. The CMC of Tween 20 is 50–60 μM [102], and that of Triton X-100 is 0.2 mM, at 25°C [98,103]. For mycolactone, the apparent saturation concentration of 1 μM determined by tensiometry (Fig 1A) may be considered equivalent to an apparent CMC. Under these conditions, the two detergents and mycolactone may behave differently at an effective (active) concentration of 60 nM. Thus, to maintain a constant ratio between the effective concentration injected in the subphase and the CMC, we further investigated the effect of each detergent on monolayer stability with an “effective concentration/CMC ratio” of 0.06 (*i*.*e*., 60 nM divided by 1 μM, as for mycolactone).

To respect this new experimental constraint, Tween 20 or Triton X-100, at final concentrations of 3.6 μM and 12 μM, respectively, were injected in the subphase of the mixture 1 monolayer (Fig 8B). The use of these new detergent concentrations had no significant effect on interaction kinetics. The main difference concerned the formation of condensed domains, which occurred more rapidly at this constant “effective concentration/CMC ratio” of 0.06 than following the injection of a 60 nM solution: 1h30 for Tween 20 (Fig 8C, row d) and 1h40 for Triton X-100 (Fig 8C, row e). However, these times remains longer than the 15 min for lipid segregation triggered by mycolactone with the same constant ratio of 0.06 (Fig 8C, row a). At an “effective concentration/CMC ratio” of 0.06, at which each molecule acts as a monomer, the same effects on lipid morphology were observed by BAM for Tween 20, Triton X-100 and mycolactone. Under these conditions, the toxin penetrated and destabilized the monolayer just like the detergents, but more efficiently, acting as a reshaping agent [97,99]. As shown in this study, mycolactone preferentially binds to monolayers containing cholesterol, and this interaction induces a destabilization of the L_o_ phase by fluidizing the monolayer, modifying the preferential interactions between SM and cholesterol, like a detergent, but not like Triton X-100 [88,97]. Similar results were recently reported for glycyrrhizin, a molecule of the saponin class extracted from plants and recognized as a natural detergent, which causes membrane perturbations after its migration toward SM/sterol-enriched membrane domains [79].

## Discussion

Mycolactones form a family of highly related macrocyclic polyketides identified as the primary virulence factors responsible for Buruli ulcer (BU), a neglected tropical disease of the skin and subcutaneous tissue caused by the environmental human pathogen *Mycobacterium ulcerans* [4]. Despite the wealth of research describing the pathogenic mechanism [14,34,37], there is still no molecular explanation of the necrosis seen in the ulcers, over and above cytopathic activity, and the immunomodulatory or analgesic properties of mycolactone [13,24]. The effects of the toxin on the cell plasma membranes they cross have never been described.

In this study, we investigated, for the first time, the membrane-binding properties of mycolactone, with Langmuir monolayers, which were used as membrane models for the fine analysis of membrane binding kinetics. We chose to use this system because its experimental design is simple and it can be adapted for investigations of the molecular insertion properties of membranotopic molecules. We studied monolayers with a lipid composition of 39% POPC, 33% SM, 9% POPE and 19% cholesterol (% mol), which was considered representative of the plasma membrane [72–75]. These monolayers were compressed at an initial surface pressure (π_i_) of 30 mN/m, a value considered representative of the lateral pressure of biological membranes [46,78]. The results obtained provided new insight into the mechanism of membrane interaction and the effect of the toxin on membrane lipid organization.

By studying the behavior of the pure toxin at an air/buffer interface, we found that mycolactone, like detergents, has surfactant properties, with an apparent surface saturation concentration of 1 μM, after which, the toxin is no longer in the monomer form (Fig 1). Experiments were conducted at a final concentration of 60 nM, to prevent artifacts during monolayer investigations. At this concentration, mycolactone interacts with the membrane as a monomer. The concentrations of mycolactone naturally present in the lesions during the course of the disease and leading to progressive ulceration are not always known. Determinations of the concentrations of mycolactone A/B in the various untreated pre-ulcerative nodules and plaques, ulcers and edematous lesions in *M*. *ulcerans*-infected human skin (biopsies) have revealed considerable variability [104]. The median concentration in all types of lesions or within the lesion itself (center or periphery) varied from 35 nM (*i*.*e*., 26 ng/mL) for pre-ulcerative lesions or the periphery of the lesions, to 596 nM (*i*.*e*., 443 ng/mL) in ulcers or 1.2 μM (*i*.*e*., 895 ng/mL) in edematous lesions. Mycolactone can also be detected in ulcer exudates obtained non-invasively from wound swabs, at concentrations of 67–270 nM (*i*.*e*., 50–200 ng/mL) [105]. Finally, it has been shown that the toxin concentration rapidly increases following inoculation with *M*. *ulcerans* in a mouse model of disease, from 385 ± 142 nM (*i*.*e*., 286 ± 105 ng/mL) on day 3, to 1.28 ± 0.29 μM (*i*.*e*., 948 ± 215 ng/mL) on day 7 and 4.85 ± 0.63 μM (*i*.*e*., 3603 ± 478 ng/mL) on day 62 [106]. Analyses of the ability of low biological concentrations (60 nM) of mycolactone to interact with the membrane provide information about the initial effects of the toxin on the plasma membrane, the first barrier that the toxin must cross to reach its intracellular targets. Presumably, these effects are progressively amplified during disease development, with the gradual increase in mycolactone concentration.

Mycolactone can bind to membranes regardless of their lipid composition (Fig 5). However, the presence of cholesterol promotes toxin insertion into the monolayer (Fig 6), highlighting the key role of this sterol in the interaction of the toxin with the membrane. Cholesterol modulates membrane fluidity and influences the organization of other lipids by changing their ordering, available area and the formation of domains of characteristic composition [107]. We also found that mycolactone had a strong effect on lateral lipid segregation in the membrane and the formation of distinct domains in monolayers containing cholesterol (Figs 5 and 7).

In the monolayer with a composition resembling that of the plasma membrane (mixture 1), preferential interactions between cholesterol (19%) and one high-melting lipid, SM (33%), which were mixed with the low-melting lipids POPC (39%) and POPE (9%) [65,90,108], led to L_o_/L_d_ segregation (Figs 3A and 7) mimicking the lateral heterogeneity of cell membranes, with the coexistence of ordered and non-ordered lipid domains [66,79,81–83,87–89,92,109,110]. This lateral heterogeneity, with the coexistence of L_d_ and L_o_ phases, would compartmentalize cellular membranes and play a key role in the lateral segregation of various classes of membrane proteins to facilitate the various cellular functions and processes occurring at the membrane [81,92,108,111–113].

We show here that mycolactone acts as a reshaping agent at very low concentrations and that, in its monomer form, it disturbs lipid segregation in monolayers (Figs 3A, 7 and 8). A similar effect has been described for penetratin, a cell-penetrating peptide known to cross cell membranes. Penetratin recruits specific lipids locally for the formation of fluid membrane patches dispersed within ordered domains [114]. The reshaping induced by mycolactone may have a direct effect on the cellular functions modulated by lipid domains, by affecting the formation of these domains. This hypothesis is supported by the promotion of mycolactone/lipid interactions within the monolayer by cholesterol and by results concerning the membrane-fluidizing effect of the toxin preventing SM aggregation (in the absence of cholesterol, Fig 4A).

Using a fluorescent derivative with a biological activity one tenth that of mycolactone, Snyder & Small showed, in 2003, that mycolactone was localized in the cytosol of murine fibroblasts cultured *in vitro* [11,12]. Similar results have been obtained with human epithelial cells and lymphocytes exposed to a ^14^C-labeled form of the toxin; the mycolactone accumulates in the cytoplasm, and not in cell plasma or nuclear membranes [13]. Overall, these results are consistent with non-cell-specific passive diffusion of the toxin through the plasma membrane to reach its intracellular targets. We show here that mycolactone, in its monomer form (*i*.*e*., 60 nM final concentration), can modify membrane lipid organization by reversing lipid segregation. Thus, if it crosses the plasma membrane at concentrations below its apparent CMC, mycolactone may disturb the natural distribution of lipids in the membrane and the formation of lipid nanodomains, with consequences for metabolic pathways involving ordered membrane domains.

As mentioned in the introduction, most of cellular targets of mycolactone are membrane protein receptors residing in ordered plasma membrane nanodomains, where their functionalities can be modulated. Mycolactone can bind and modulate the activity of two members of a family of scaffold proteins, Wiskott-Aldrich syndrome protein (WASP) and neural WASP (N-WASP), which transduce various endogenous signals through a dynamic remodeling of the actin cytoskeleton [17]. However, sphingolipid-cholesterol domains have been shown to be the preferred platforms for membrane-linked actin polymerization mediated by *in situ* phosphatidylinositol 4,5-bisphosphate (PIP_2_) synthesis and tyrosine kinase signaling through the WASP-ARP2/3 pathway (PIP_2_ stimulates *de novo* actin polymerization by activating the pathway involving WASP and the actin-related protein complex ARP2/3 [115]). Mycolactone binding to WASP involves a lysine-rich basic region (BR) [17] that has also been implicated in the activation of WASP/N-WASP by PIP_2_ in both the allosteric and the oligomerization domains [116]. The L_o_ phase reversion triggered by the membrane insertion of mycolactone may therefore play an important role in modulating the WASP-Arp 2/3 pathway. Could mycolactone replace PIP_2_ in the disorganized sphingolipid-cholesterol microdomains and bind to WASPs?

Mycolactone also triggers a depletion of thrombomodulin (TM) receptors on the surface of endothelial cells, leading to disruption of the protein C anticoagulation pathway. This depletion has been observed *in vitro* in human dermal microvascular endothelial cells (HDMVECs) exposed to a very low dose (2 ng/mL *i*.*e*., 2.69 nM) of mycolactone and, *in vivo*, in the subcutaneous tissues of punch biopsies, and is strongly associated with the fibrin deposition commonly observed in BU skin lesions [34]. Nevertheless, TM receptors for protein C activation and activated protein C (APC) signaling pathways are colocalized in the lipid microdomains of endothelial cells [35,36]. This localization in the same domain is the key requirement for APC signaling pathways in endothelial cells [35,36]. Again, the change in lipid segregation, in addition to the observed depletion of TM receptors, due to the interaction of mycolactone with the plasma membrane during its passage into the cell, may disturb lipid microdomain formation in the membrane, thereby modifying the various signaling pathways requiring lipid membrane domains as signaling platforms [111]. Could the TM depletion observed in endothelial cells exposed to mycolactone [34] also be due to changes in lipid segregation in the plasma membrane, to which this receptor is targeted for functional activity?

Finally, many of the pathogenic effects of mycolactone could potentially be explained by a blockade of protein translocation [14]. Nevertheless, no unifying molecular mechanism underlying the pleiotropic actions of mycolactone has yet been identified. It remains, unclear, for example, how mycolactone blocks ER translocation at the molecular level? Baron *et al*. recently showed that mycolactone targets the α subunit of the Sec61 translocon, thereby strongly blocking the production of secreted and integral membrane proteins [38]. However, it is now widely accepted that the signal sequence of secreted proteins is clipped off during translation by a signal peptide peptidase (SPP). After chaperone-assisted folding, the mature protein is then released into the lumen of the ER immediately after its synthesis [41]. However, it has been suggested that SPP cleavage may be regulated through the control of substrate entry into the microdomains of the membrane containing SPP [117,118]. During the fractionation of rough ER integral membrane proteins with 0.18% Triton X-100, most ER-resident proteins, and the Sec61alpha subunit in particular, were found to be present in the microdomain-like fraction [44]. Little is known about the role of cholesterol in basic ER functions, but recent studies have clearly suggested that cholesterol may act with ER membrane proteins to regulate several important functions of the ER, including the folding, degradation, compartmentalization, and segregation of ER proteins, and sphingolipid biosynthesis [119]. McKenna *et al*. have provided biochemical evidence that mycolactone induces a conformational change in the transmembrane pore-forming Sec61alpha subunit of the translocon [39]. Hence, taking into account the potentially important contribution of cholesterol to the effect of mycolactone on membrane lipid segregation, we cannot exclude the possibility that the ER membrane may also be reshaped by mycolactone, abolishing the functions of the ER membrane-resident proteins. In the case of the Sec61 translocon, this hypothesis is consistent with the induction of a stabilized closed conformation of the Sec61alpha unit (forming the central gated protein-conducting channel across the ER membrane) [39,45] mediated by lipid redistribution in microdomains to facilitate the interaction of mycolactone close to the luminal plug of Sec61alpha, as recently suggested by Baron *et al*. [38].

The redistribution of lipid microdomains from the plasma membrane to mitochondria has recently been demonstrated in other contexts and diseases and is robust to this hypothesis [120]. The alteration of ER lipid microdomains initiates lipotoxicity in pancreatic β-cells, disturbing protein trafficking and initiating ER stress, thereby contributing to type 2 diabetes [121]. In the same way, the disruption of lipid microdomains stimulates phospholipase D activity in human lymphocytes, and this activation conveys antiproliferative signals in lymphoid cells, by impairing the transduction of mitogenic signals [122]. The gastrointestinal symptoms observed in patients suffering from Niemann-Pick type C disease are the consequence of changes in the composition of membrane lipid domains, resulting in impaired trafficking and an apical sorting of the major intestinal disaccharidases to the plasma membrane with a decrease in their functional capacities [123,124]. Edelfosine, an alkylphospholipid analog (APL) belonging to a family of synthetic antitumor compounds, induces apoptosis in several hematopoietic cancer cells, by targeting various subcellular structures at the membranes [125,126]. By recruiting death receptor and downstream apoptotic signaling molecules to ordered lipid domains, it displaces survival signaling molecules from these membrane domains. Edelfosine-induced apoptosis in solid tumor cells is mediated by an ER stress response and evidence has been obtained *in vitro* and *in vivo* to suggest that edelfosine treatment induces a redistribution of lipid domains from the plasma membrane to mitochondria, suggesting a raft-mediated link between the plasma membrane and mitochondria [120]. All these examples suggest that membrane reshaping can occur in different diseases, and that the disturbance of lipid segregation observed here with mycolactone is not an isolated case.

In summary, all the cellular targets of mycolactone are membrane-bound proteins, and, with the exception of the Sec61 translocon, all are known to be regulated by ordered microdomains, which provide a platform for the assembly of signaling complexes and prevent cross-talk between pathways [25,110]. By disturbing lipid segregation in membranes containing cholesterol, mycolactone affects many cell functions and signaling pathways. This membrane remodeling may occur in synergy with the previously demonstrated effects of mycolactone on its intracellular targets, possibly even potentiating these effects. It is tempting to speculate that microdomain remodeling in membranes underlies the molecular events *via* which mycolactone affects multiple targets, but further studies are required to confirm this.

## Materials and methods

### Chemicals

Ultrapure water was obtained from a PURELAB option Q7 system (VEOLIA WATER STI, France). Phosphate-buffered saline (PBS, 2.8 mM KCl, 140 mM NaCl and 10 mM phosphate, pH 7.40 ± 0.05 at 25°C) was prepared by dissolving tablets purchased from AppliChem GmbH (Darmstadt, Germany) in ultrapure water.

We obtained 1-palmitoyl-2-oleoyl-*sn*-glycerophosphocholine (POPC), 1-palmitoyl-2-oleoyl-*sn*-glycerophosphoethanolamine (POPE), cholesterol (Chol) from ovine wool (≥98%) and 23-(dipyrrometheneboron difluoride)-24-norcholesterol or TopFluor Cholesterol from Avanti Polar Lipids (Alabaster, Alabama, USA). Sphingomyelin (SM) from chicken egg yolk (≥95%) was purchased from Sigma-Aldrich (Saint-Quentin Fallavier, France). All chemicals were used as received. The solvents were of analytical grade (Sigma-Aldrich, Saint-Quentin Fallavier, France). The lipid mixtures were prepared at a concentration of 1 mg/mL in chloroform (or chloroform/methanol, 9:1 *v*/*v*, when containing SM) and stored at -20°C under argon to prevent lipid oxidation.

Polyethylene glycol sorbitan monolaurate (Tween 20) and polyethylene glycol *tert*-octylphenyl ether (Triton X-100) were also purchased from Sigma-Aldrich (Saint-Quentin Fallavier, France).

### Mycolactone extraction and purification

Mycolactones A/B were purified from *M*. *ulcerans* extracts as previously described [6,127]. Briefly, S4018, an African strain of *Mycobacterium ulcerans* obtained from a patient in Benin, was grown in Middlebrook 7H10 agar supplemented with oleic albumin dextrose catalase growth supplement. The bacteria were resuspended in chloroform-methanol (2:1, *v*/*v*) and cell debris was removed by centrifugation. Folch extraction was performed by adding 0.2 volumes of water. The organic phase was dried and phospholipids were precipitated with ice-cold acetone. The acetone-soluble lipids were loaded onto a thin layer chromatography plate and eluted with chloroform-methanol-water (90:10:1, *v*/*v*/*v*) as the mobile phase. The yellow band with a retention factor of 0.23 was scraped off the plate, filtered, evaporated, resuspended in absolute ethanol and then stored in amber glass tubes in the dark. Its concentration was determined by measuring absorbance (λ_max_  =  362 nm, log ε  =  4.29), and its purity (>98%) was evaluated with a Shimadzu Ultra-Fast Liquid Chromatograph (UFLC XR system with a CBM-20A controller, a CTO-10AS Prominence column oven, LC-20AB pumps, an SPD M20A diode array detector (Shimadzu, Japan)) and a reverse C18 column (Zorbax 23 Eclipse XDB-C18, 9.4×250mm, Particle Size: 5 μm (Agilent, USA)).

### Langmuir monolayer experiments

Monolayers were prepared on a KSV 2000 Langmuir-Blodgett trough (3 multi-compartments, KSV NIMA, Biolin Scientific, Finland), with a symmetric compression system. The rectangular trough had a volume of 80 mL and a surface area of 119.25 cm^2^. A Wilhelmy plate attached to an electronic microbalance was used to measure the surface pressure (π), with an accuracy of ± 0.5 mN/m. The trough was cleaned with successive baths of dichloromethane, ethanol and ultrapure water, and filled with a filtered PBS solution. The subphase buffer was maintained at the desired temperature (20°C or 25°C) throughout the experiment, with an Ecoline RE106 low-temperature thermostat (LAUDA, Germany). It was not possible to work at a higher temperature, due to subphase evaporation, which can falsify surface pressure measurement during the run. Lipid mixtures in chloroform were gently spread at the air/liquid interface of the PBS subphase. The solvent was allowed to evaporate off for 15 minutes, and the monolayer was then slowly compressed by two mobile barriers at a constant rate of 0.045 nm^2^.molecule^-1^.min^-1^ until an initial surface pressure (π_i_) of 5 to 30 mN/m was reached. A lag time of about 1 h was then applied to allow the monolayer to relax and stabilize. The surface area was then kept constant by stopping the movement of the mobile barriers.

Mycolactone (1 mg/mL in ethanol) was injected (4.45 μL) into the subphase just beneath the lipid monolayer at a final concentration of 60 nM. The changes in surface pressure induced by the interaction of mycolactone with the monolayer were recorded continually, as a function of time, with a computer-controlled Langmuir film balance KSV NIMA (Biolin Scientific, Finland), until the equilibrium surface pressure (π_e_) was reached, indicating the end of the adsorption process. All measurements were repeated at least three times for each set of conditions, with a satisfactory reproducibility, and the mean values are reported here.

We used a monolayer with a lipid concentration closely resembling that of the plasma membrane, according to several authors [72–75]. This monolayer contained 39% phosphatidylcholine (POPC), 33% sphingomyelin (SM), 9% phosphatidylethanolamine (POPE) and 19% cholesterol (Chol) (in mol%). For evaluation of the influence of cholesterol on both the membrane-binding capacity and effects of mycolactone on phospholipid membrane organization, we also analyzed monolayers with a different composition devoid of cholesterol but with the molar ratios of the other lipids maintained. This second monolayer contained 48% POPC, 41% SM, and 11% POPE.

### Adsorption kinetics of the mycolactone

The surface pressure increase (Δπ in mN/m) after the mycolactone injection corresponds to π_e_ - π_i_. The curve of surface pressure increase (Δπ) as a function of time (*t*) recorded during the adsorption of mycolactone onto lipid monolayers corresponds to the adsorption kinetics of the molecule.

### Determination of the binding parameters of mycolactone

The parameters characterizing the binding of mycolactone to different lipid membranes were further determined, as previously described [50,56,93,94]. Briefly, Δπ was plotted against different initial surface pressures π_i_ to determine: i) the critical surface pressures π_c_, also known as the maximum insertion pressure (MIP), which is calculated by extrapolation of the linear regression line to the *x*-axis (Δπ_max_ = 0) and, ii) the synergy factor, *a*, measured by adding 1 to the slope obtained from the linear regression of Δπ as a function of π_i_. The uncertainty on MIP and the synergy factor, *a*, were determined as previously described [50,94]. The uncertainty on MIP was calculated with a 95% confidence interval from the covariance of the experimental data for the linear regression [50]. The uncertainty on synergy was calculated as previously described [94]. These experimental errors were directly determined with the free binding parameters calculator software (http://www.crchudequebec.ulaval.ca/BindingParametersCalculator) developed by Salesse’s group.

### Brewster angle microscopy

Brewster angle microscopy (BAM) was used to characterize the lipid domain morphology of monolayers at the air/water interface [62,64]. Monolayer morphology was determined before and after mycolactone injection, with an EP^3^SW Brewster angle microscope (Accurion, Germany) equipped with a 532 nm laser, a polarizer, an analyzer and a CCD camera. BAM image size was 483 × 383 μm^2^. For ultrathin films, reflectance depends on both the thickness and refractive index of the monolayer. The different views of the interfacial film were reconstituted with EP^3^viewer BAM software (Accurion, Germany), based on the brightness of the BAM pictures. For a constant refractive index, reflectance is directly linked to the thickness of the interfacial film.

### Fluorescence microscopy

Langmuir films were generated in a custom-built cylindrical Teflon trough with a quartz window, containing 25 mL of filtered buffer, connected to a peristaltic pump. The system was mounted on the stage of a Zeiss Observer Z1 microscope (Carl Zeiss Vision, Germany) for fluorescence microscopy (FM) experiments.

Samples were prepared for FM by replacing 0.5 mol% of the cholesterol with 0.5 mol% of the sterol fluorescent probe, TopFluor Cholesterol [96]. The images were acquired at excitation and emission wavelengths of 495 and 507 nm, respectively. Images were processed and analyzed with dedicated Zeiss software (Axio Vision 4.8).

We used a Hamilton syringe to spread a few microliters of a 1 mg/mL phospholipid solution in chloroform onto the buffer subphase until the desired π_i_ was reached. One hour later, after the solvent had evaporated and the lipid monolayer had stabilized at the desired initial surface pressure (π_i_), the mycolactone A/B solution was injected, with a Hamilton syringe, into the subphase through the lipid monolayer, with gentle stirring. During the time course of the experiment, changes in surface pressure (π) were also recorded simultaneously and continuously with a KSV NIMA computer-controlled Langmuir film balance.

## Supporting information

S1 Fig **Chemical structure of mycolactone A/B showing the cyclic lactone core (C1-C11) and the two polyketide side chains, *i*.*e*., the C12-C20 “Northern” chain and the longer C1’-C16’ “Southern” chain.** The most virulent strains of *M*. *ulcerans* generate mycolactone A/B (Z-/E-isomers of the C4’-C-5’ bond in the long “Southern” polyketide side chains) in a 3:2 ratio, and most research into the biological functions of this toxin has focused on mycolactone A/B [15].
(TIF)

S2 Fig **Surface pressure (π)–molecular area (A) isotherms of pure POPC and mixed monolayers recorded at 20° or 25°C.** All the monolayers were prepared with the same chloroform solutions. The compositions are given in mol%. Mixture 1 corresponded to 39% POPC, 33% SM, 9% POPE, 19% Chol. Mixture 2 corresponded to 48% POPC, 41% SM, 11% POPE. Isotherms were recorded on PBS subphase (pH 7.4) at a constant compression rate of 0.045 nm^2^.molecule^-1^.min^-1^. The estimated error is ±0.05 mN/m for π and ≤ 0.01 nm^2^ for (A). Each isotherm corresponds to the mean of three experiments. The isotherms of POPC, recorded at 20 or 25°C, are consistent with a liquid-expanded (LE) phase for all compression values and can be superimposed, consistent with the T_m_ value (-4°C) of POPC. The molecular area at collapse (A_coll_ = 40 mN/m) is close to the expected value of ~40 Å^2^/molecule for two acyl chains. A similar area is obtained for the 81% POPC/ 19% cholesterol mixture at 25°C. This area decreases to ~32 Å^2^ at 20°C. The values obtained for the monolayers of mixtures 1 and 2 formed with the same solution of POPC were lower than those for pure POPC, due to the condensing effect of Chol and high-melting lipids, such as SM (T_m_ = 41.4°C), present in mixtures 1 and 2, respectively. These isotherms illustrate the effects of temperature and cholesterol in the mixed monolayers.
(TIF)

S3 Fig Stability of model lipid monolayers at the working surface pressure. Changes in surface pressure π as a function of time after the injection of ethanol (the solvent for mycolactone) beneath the monolayer consisting of (A) mixture 1 (39% POPC, 33% SM, 9% POPE, 19% Chol) or (B) mixture 2 (48% POPC, 41% SM, 11% POPE) at 20°C (solid line) or 25°C (dashed line). Ethanol was injected into the PBS subphase (pH 7.4) after a relaxation time of one hour (arrows). Surface area was kept constant during the run. Each measurement was performed at least three times for each condition, and a representative curve is presented here.
(TIF)

S4 Fig Effect of mycolactone on the lipid organization of mixed monolayers in the presence of cholesterol. BAM images of 39% POPC, 33% SM, 9% POPE, 19% Chol (mixture 1) monolayers **without ethanol injection** (row a) or after the injection (4.45 μL) of mycolactone **at a final concentration of 1 μM** (row b) into the PBS subphase (pH 7.4) beneath the interfacial film compressed at an initial surface pressure of 30 mN/m at 20°C. The injection was performed after a relaxation time of one hour, and the surface area was kept constant (mobile barriers were stopped). Image scale: 483 × 383 μm^2^.
(TIF)

S5 Fig π-*A* isotherm of a pure sphingomyelin monolayer. The isotherm was recorded on PBS subphase (pH 7.4). (A) & (B): BAM images were recorded during compression of the monolayer at a constant rate of 0.045 nm^2^.molecule^-1^.min^-1^. The estimated error is ± 0.05 mN/m for π and ≤ 0.01 nm^2^ for A. LE: liquid expanded; LC: liquid condensed. Image scale: 483 × 383 μm^2^.
(TIF)

S1 Appendix Mycolactone aggregation state determination by DLS. (DOCX)
