# Supplementary figures and images for: The potent effect of mycolactone on lipid membranes
Source: PLoS Pathog. 2018 Jan 10;14(1):e1006814. doi: 10.1371/journal.ppat.1006814 (PMC5779694; doi:10.1371/journal.ppat.1006814)

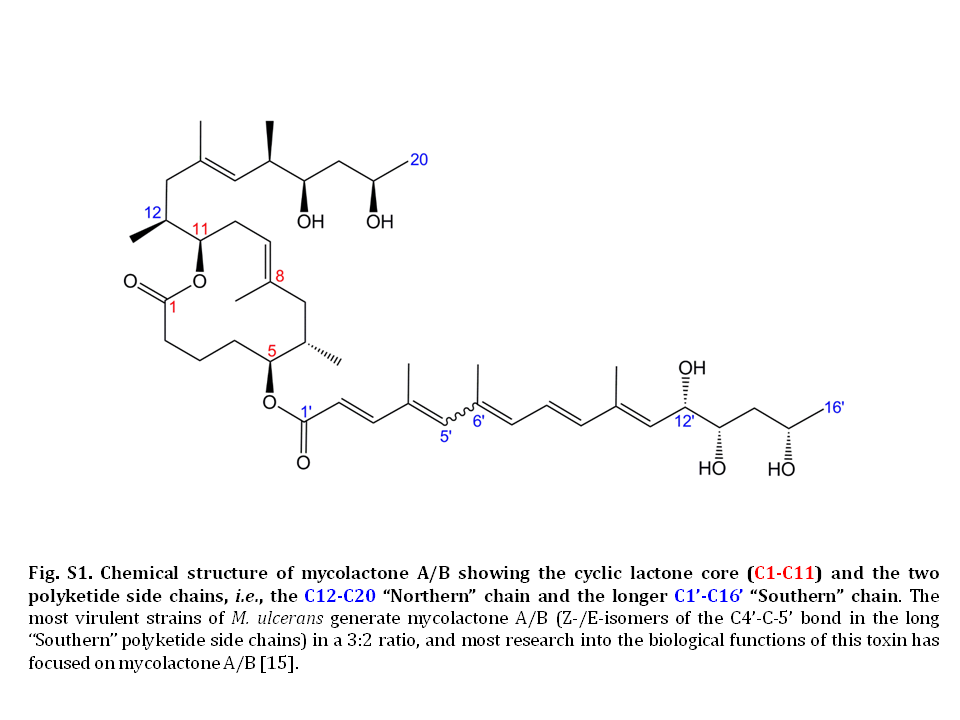

Supplement: S1 Fig — Chemical structure of mycolactone A/B showing the cyclic lactone core (C1-C11) and the two polyketide side chains, i.e., the C12-C20 “Northern” chain and the longer C1’-C16’ “Southern” chain. The most virulent strains of M. ulcerans generate mycolactone A/B (Z-/E-isomers of the C4’-C-5’ bond in the long “Southern” polyketide side chains) in a 3:2 ratio, and most research into the biological functions of this toxin has focused on mycolactone A/B [15]. (TIF) [file ppat.1006814.s001.tif]

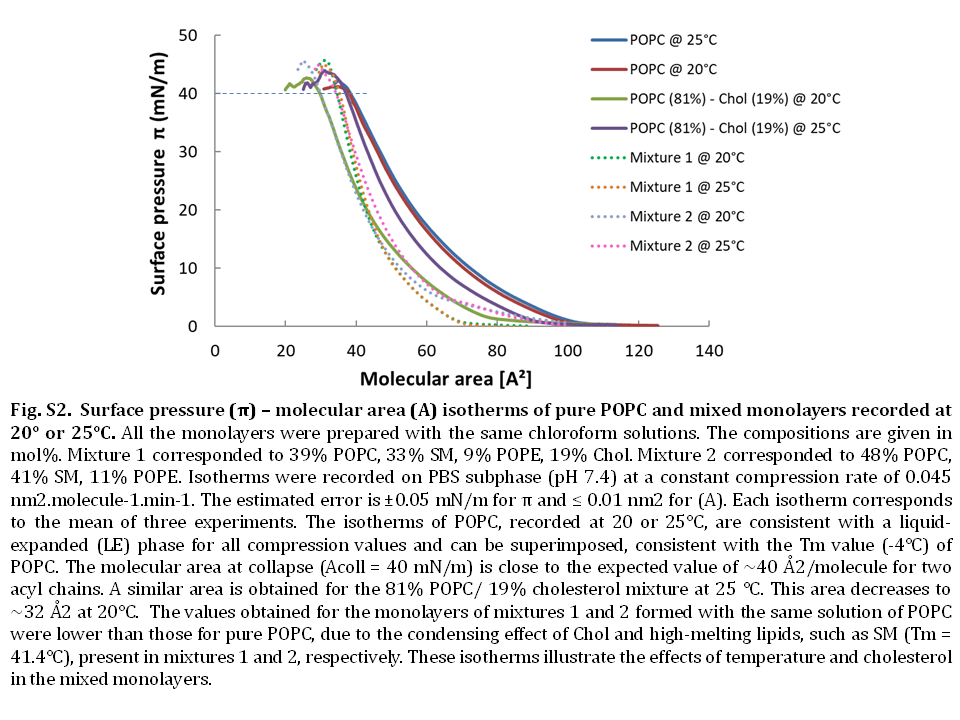

Supplement: S2 Fig — Surface pressure (π)–molecular area (A) isotherms of pure POPC and mixed monolayers recorded at 20° or 25°C. All the monolayers were prepared with the same chloroform solutions. The compositions are given in mol%. Mixture 1 corresponded to 39% POPC, 33% SM, 9% POPE, 19% Chol. Mixture 2 corresponded to 48% POPC, 41% SM, 11% POPE. Isotherms were recorded on PBS subphase (pH 7.4) at a constant compression rate of 0.045 nm2.molecule-1.min-1. The estimated error is ±0.05 mN/m for π and ≤ 0.01 nm2 for (A). Each isotherm corresponds to the mean of three experiments. The isotherms of POPC, recorded at 20 or 25°C, are consistent with a liquid-expanded (LE) phase for all compression values and can be superimposed, consistent with the Tm value (-4°C) of POPC. The molecular area at collapse (Acoll = 40 mN/m) is close to the expected value of ~40 Å2/molecule for two acyl chains. A similar area is obtained for the 81% POPC/ 19% cholesterol mixture at 25°C. This area decreases to ~32 Å2 at 20°C. The values obtained for the monolayers of mixtures 1 and 2 formed with the same solution of POPC were lower than those for pure POPC, due to the condensing effect of Chol and high-melting lipids, such as SM (Tm = 41.4°C), present in mixtures 1 and 2, respectively. These isotherms illustrate the effects of temperature and cholesterol in the mixed monolayers. (TIF) [file ppat.1006814.s002.tif]

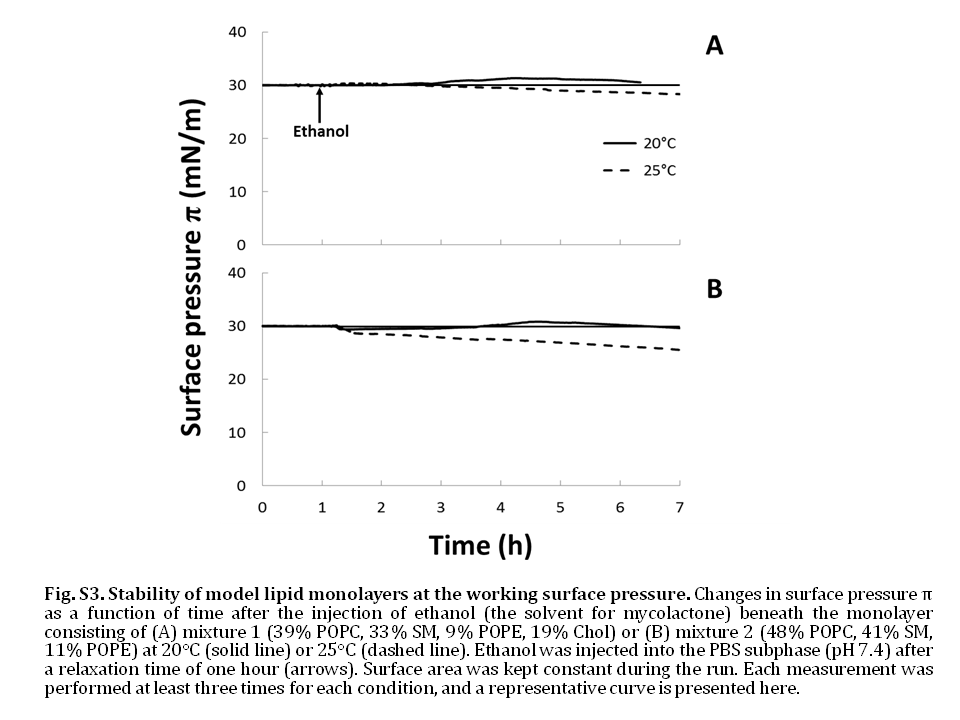

Supplement: S3 Fig — Changes in surface pressure π as a function of time after the injection of ethanol (the solvent for mycolactone) beneath the monolayer consisting of (A) mixture 1 (39% POPC, 33% SM, 9% POPE, 19% Chol) or (B) mixture 2 (48% POPC, 41% SM, 11% POPE) at 20°C (solid line) or 25°C (dashed line). Ethanol was injected into the PBS subphase (pH 7.4) after a relaxation time of one hour (arrows). Surface area was kept constant during the run. Each measurement was performed at least three times for each condition, and a representative curve is presented here. (TIF) [file ppat.1006814.s003.tif]

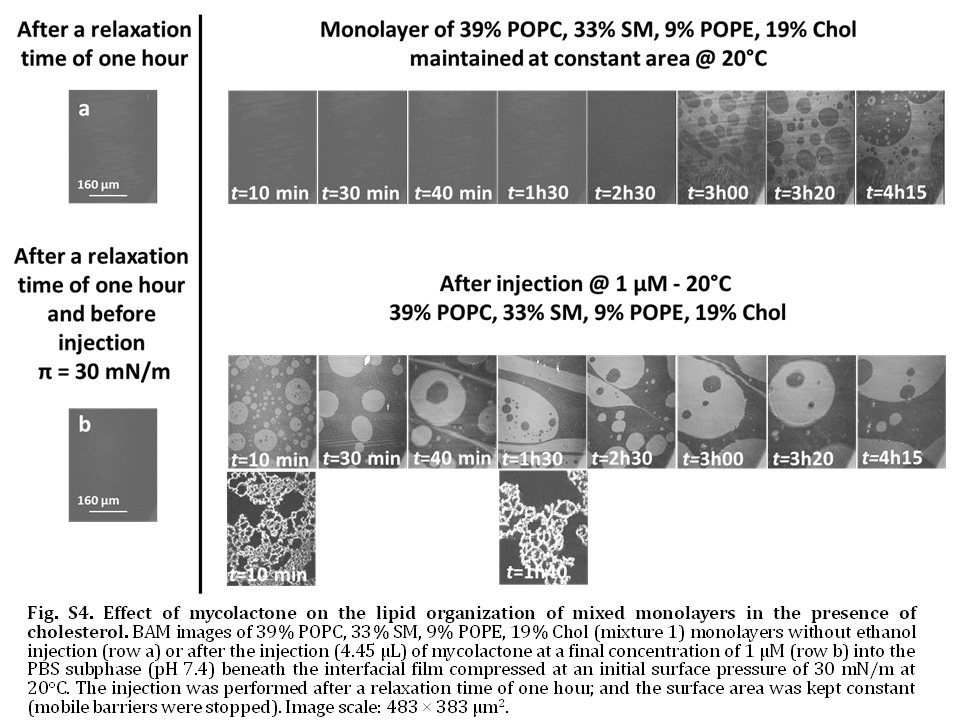

Supplement: S4 Fig — BAM images of 39% POPC, 33% SM, 9% POPE, 19% Chol (mixture 1) monolayers without ethanol injection (row a) or after the injection (4.45 μL) of mycolactone at a final concentration of 1 μM (row b) into the PBS subphase (pH 7.4) beneath the interfacial film compressed at an initial surface pressure of 30 mN/m at 20°C. The injection was performed after a relaxation time of one hour, and the surface area was kept constant (mobile barriers were stopped). Image scale: 483 × 383 μm2. (TIF) [file ppat.1006814.s004.tif]

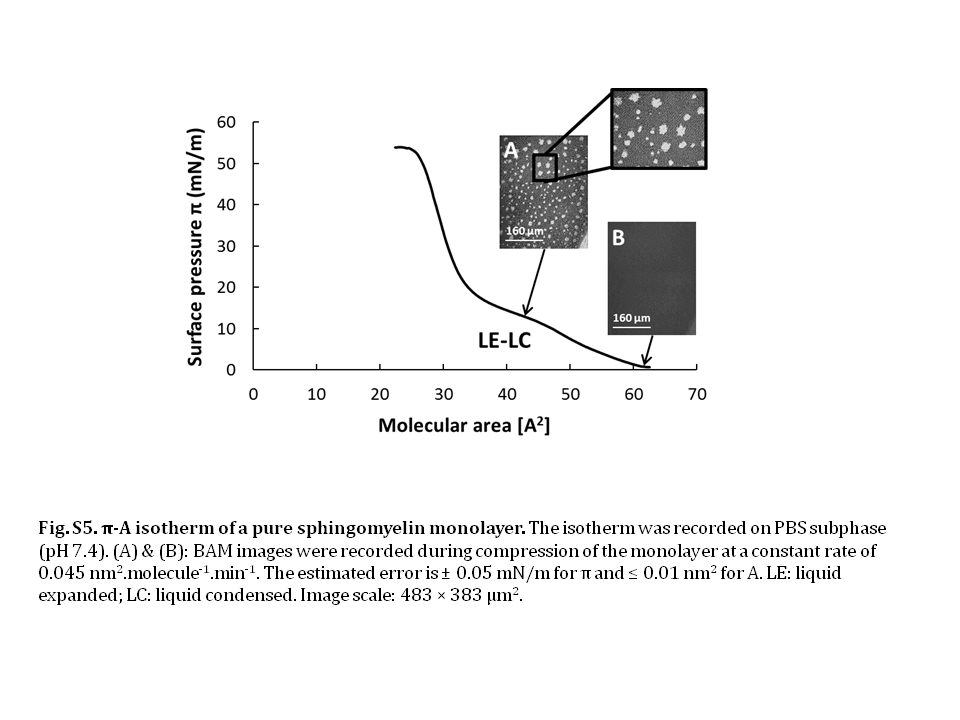

Supplement: S5 Fig — The isotherm was recorded on PBS subphase (pH 7.4). (A) & (B): BAM images were recorded during compression of the monolayer at a constant rate of 0.045 nm2.molecule-1.min-1. The estimated error is ± 0.05 mN/m for π and ≤ 0.01 nm2 for A. LE: liquid expanded; LC: liquid condensed. Image scale: 483 × 383 μm2. (TIF) [file ppat.1006814.s005.tif]
