# Supplementary material for: The potent effect of mycolactone on lipid membranes
Source: PLoS Pathog. 2018 Jan 10;14(1):e1006814. doi: 10.1371/journal.ppat.1006814 (PMC5779694; doi:10.1371/journal.ppat.1006814)
Supplement: S1 Appendix — (DOCX) [file ppat.1006814.s006.docx]

**Appendix S1: Mycolactone aggregation state determination by DLS**

**Materials & Methods**

*Dynamic Light Scattering*

Dynamic light scattering (DLS) was used to determine the hydrodynamic diameter (D_h_) of mycolactone with a Zetasizer Nano ZS (Malvern Instruments) experimental setup and a scattering angle of 173°. The software supplied by the manufacturer determines particle size, by using the Stokes-Einstein relationship to obtain the intensity-averaged size distribution from the raw correlation data. For the mycolactone analysis, size distribution assays were performed at 25°C in a volume of 50 µL in ZEN0040 cuvettes. For each assay, three measurements were performed, consisting of 10-15 runs of 10 seconds each. The results are displayed as correlograms, Z-average (mean intensity-averaged size) and Di50 mean intensity size determined with Zetaziser software version 7.12. Mycolactone was prepared at several concentrations in PBS pH 7.4: 60 nM, 0.5 µM, 1 µM, 5 µM and 10 µM.

**Results and discussion**

*Hydrodynamic diameter of mycolactone*

We used DLS to observe the aggregation state of mycolactone in PBS, pH 7.4. The DLS technique is ideal for detecting large diffusing molecules. The zetasizer can detect molecules from 0.6 nm to 10 µm. We show here that the signal obtained at 60 nM is below the acceptable value of signal-to-noise ratio (intercept) to confirm an aggregation at this low concentration (Table AS1). Signal-to-noise ratios of ≥ 0.3 to 0.9 are considered acceptable. The 60 nM signal was similar to that for PBS, as shown by the correlogram (Fig. AS1A). However, a diffusion signal is clearly visible with higher concentrations of mycolactone, from 0.5 µM to 10 µM. The signal-to-noise ratio is in the acceptable range (Table AS1). The slow decay of the signal in the correlogram at approximately 100 µs is indicative of large slow-moving molecules (Fig. AS1A). The extended curve of the correlogram between 100 µs and 1000 µs suggests that the population is not homogeneous, as confirmed by the dispersity in Table AS1. The least dispersed concentration was 10 µM. The software applied the Stokes-Einstein relationship to the translation diffusion of intensity from the molecules to obtain the intensity-averaged size distribution. The calculated mean intensity-averaged size (Z.average) shows that the hydrodynamic diameter of mycolactone ranges from 200 to 326 nm (Table AS1). The distribution of size as a function of intensity (Fig. AS1B), clearly indicates that mycolactone forms large diffusing aggregates at concentrations as low as 0.5 µM. Fig. AS1B shows a concentration-dependent increase in intensity, suggesting that size remains constant at higher concentrations. We conclude that mycolactone forms spheres with an approximate size of 200 nM, in solution.

**
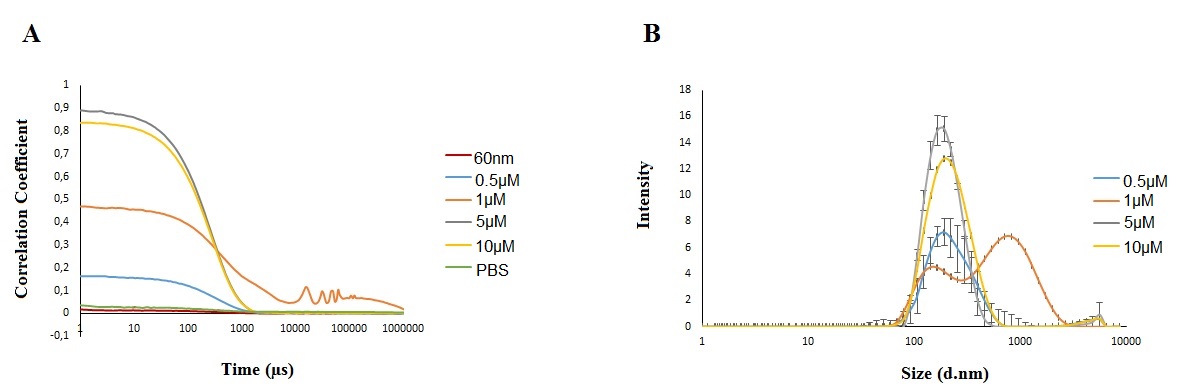
**

**Figure AS1:** Mycolactone size distribution at 25°C. **A**) Correlogram for increasing concentrations of mycolactone. **B)** Mycolactone size distribution as a function of intensity at different concentrations.

**Table AS1: Size distribution at 25°C. #** Three measurements for each molecule**. Intercept** signal-to-noise ratio (0-1)**. Z.average d.nm** mean intensity-averaged size, corresponding to diameter. **Pdi** dispersity, providing an indication of the heterogeneity of the sample (0-1). **Di50** diameter for the intensity corresponding to 50% of the sample. NA: not applicable

| **Mycolactone concentration** | **Intercept #** | **Z-average d.nm #** | **Pdi #** | **Di50 #** |
| --- | --- | --- | --- | --- |
| PBS | 0.153±0.019 | NA | NA | NA |
| 60 nM | 0.108±0.008 | NA | NA | NA |
| 0.5 µM | 0.446±0.04 | 240.83±6.7 | 0.223±0.05 | 281.33±10.21 |
| 1 µM | 0.598±0.03 | 326.3±13.47 | 0.431±0.12 | 510.66±66.19 |
| 5 µM | 0.942±0.003 | 192.9±18.11 | 0.224±0.12 | 186.66±7.23 |
| 10 µM | 0.920±0.004 | 197.26±3.6 | 0.186±0.02 | 208±9.53 |
